# Optimization of automatic generation controllers in renewable multi-area power systems using the Fata Morgana algorithm

**DOI:** 10.1038/s41598-025-27191-7

**Published:** 2025-12-08

**Authors:** Aykut Fatih Güven, Erdinç Şahin, Onur Özdal Mengi, Mohit Bajaj, Viktoriia Bereznychenko

**Affiliations:** 1 Department of Electrical and Electronics Engineering, Yalova University, Yalova, Turkey; 2 Department of Computer Engineering, Giresun University, Giresun, Turkey; 3 Department of Electrical and Computer Engineering, University of New Brunswick, Fredericton, Canada; 4 Department of Electrical and Electronics Engineering, Giresun University, Giresun, Turkey; 5 Department of Electrical Engineering, Graphic Era (Deemed to be University), Dehradun, 248002 India; 6 Hourani Center for Applied Scientific Research, Al-Ahliyya Amman University, Amman, Jordan; 7 Graphic Era Hill University, Dehradun, 248002 India; 8 Department of Theoretical Electrical Engineering and Diagnostics of Electrical Equipment, Institute of Electrodynamics, National Academy of Sciences of Ukraine, Beresteyskiy, 56, 03680 Kyiv Ukraine

**Keywords:** FATA, Multi area power systems, Meta-heuristic algorithms, Optimization, Real-time simulation, Energy science and technology, Engineering, Mathematics and computing

## Abstract

The increasing integration of renewable energy sources introduces severe intermittency in multi-area power systems (MAPS), resulting in significant voltage and frequency fluctuations. This study addresses this problem by implementing an automatic generation control (AGC) framework for a two-area hybrid power system composed of solar, wind, and thermal units. Four types of controllers (PI, PIDn, fractional-order PI (FOPI), and predictive PIDn (PPIDn)) were optimized using four recent metaheuristic algorithms: golden jackal optimization (GJO), educational competition optimizer (ECO), escape algorithm (ESC), and the newly proposed Fata Morgana Algorithm (FATA). The results demonstrate that the FATA-optimized PIDn controller provides the best dynamic performance, achieving an ITAE value of 0.18676, which represents an improvement of over 4.6% compared to the best established optimizer (ESC). Real-time validation on the OPAL-RT OP5707 platform confirmed the practical feasibility of the proposed FATA-based control strategy, verifying its ability to enhance frequency stability. These findings highlight the novelty and efficiency of FATA in optimizing AGC parameters for renewable-based multi-area power systems.

## Introduction

Over the years, advancements in power systems have significantly increased their complexity compared to a century ago. Modern power systems operate at much higher power levels, making their control more challenging. The transition from megawatt (MW) to gigawatt (GW) power levels, the growing number and diversity of energy sources, the interconnection of regions through long transmission lines, unbalanced load distribution, voltage magnitude and phase differences between areas, fault conditions, and the integration of harmonic-generating systems such as electric vehicles have made power system operation increasingly complex^1^.

Furthermore, the intermittent nature of renewable energy sources, such as solar and wind, adds to these challenges. Since their power generation depends on environmental conditions, the output fluctuates continuously. This variability poses significant difficulties in maintaining system stability and ensuring that loads receive voltage and power at the desired frequency and amplitude. Therefore, precise voltage and frequency control is essential both within individual generation areas and across interconnected regions^2,3^.

**Fig. 1:**
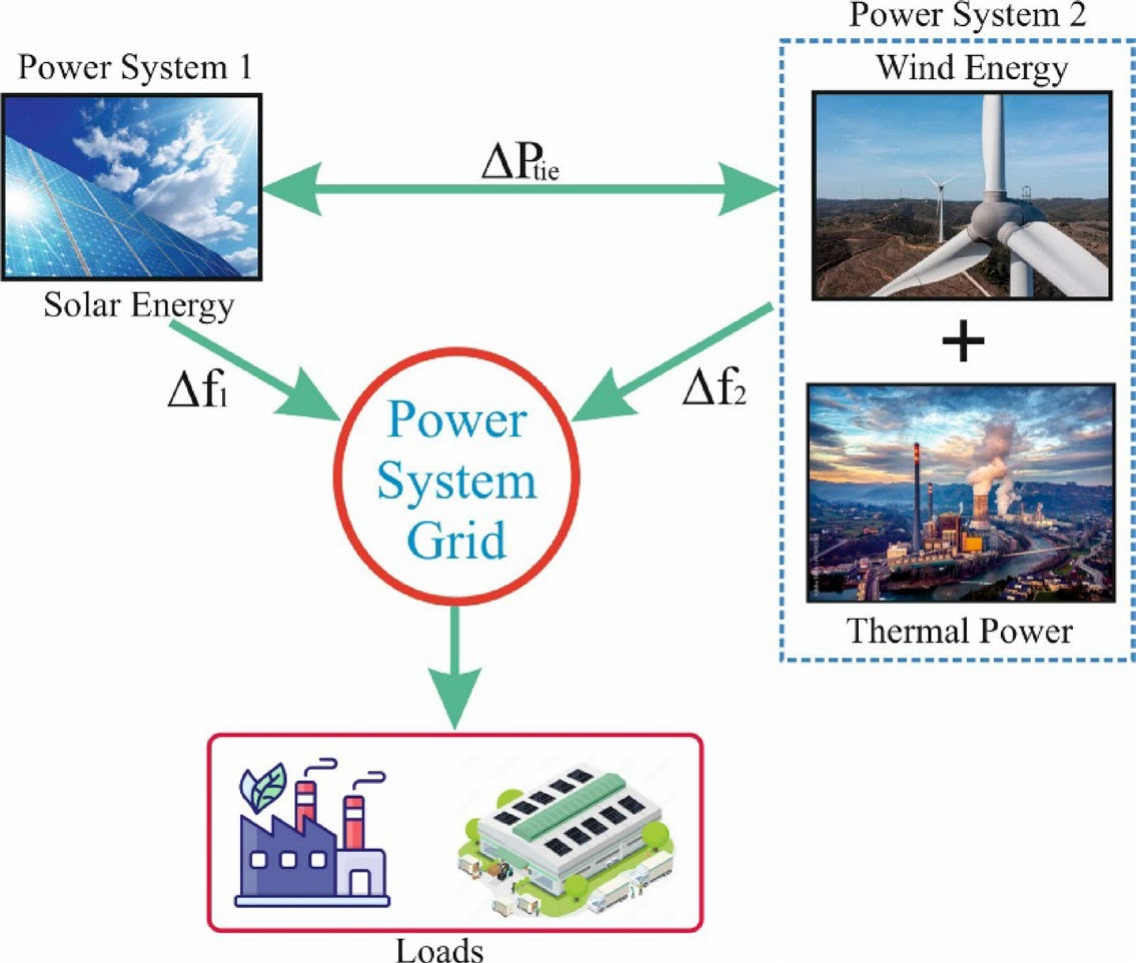
Conceptual design for TAPS.

In this study, automatic generation control (AGC), also referred to as load frequency control (LFC), is implemented in a two-area power system consisting of three different energy sources. Figure 1 illustrates the system structure, which integrates wind, solar, and thermal power generation.

In area-1, power is generated from solar energy, while area-2 consists of both wind and reheat-thermal power generation stations. The frequency deviation in area-1 is represented as Δf_1_, while in area-2, it is denoted as Δf_2_. The power deviation between the two areas is expressed as ΔP_tie_. The generated power is supplied to various energy consumers, such as industrial facilities and residential loads.

Each power generation area is controlled independently. If a region is not connected to another system, it can operate autonomously. However, when different energy generation systems are interconnected within a grid, they must remain balanced with respect to each other. To achieve this balance, various controllers are employed. Commonly used controllers include proportional-integral (PI), proportional-integral-derivative (PID)^4^, fractional order PID (FOPID)^5^, fuzzy logic controllers (FLC)^6^, and hybrid structures such as fuzzy logic integrated PID (FLPID) controllers^7,8^. The parameters of these controllers are typically tuned using different optimization algorithms. Several optimization techniques have been utilized, including the whale optimization algorithm (WOA)^9^, the gray wolf optimizer (GWO)^10^, genetic algorithm (GA) -based methods^11^, JAYA algorithm^12^, the water cycle algorithm (WCA)^13^, and chaotic butterfly optimization^14^. Intelligent and adaptive control approaches have recently gained attention for improving renewable-based power system performance. Neural network controllers have been applied for battery storage regulation under varying load profiles^15^, while hybrid energy management strategies integrating renewables and EV storage enhance grid flexibility^16^. Fuzzy-assisted sliding mode and neuro-fuzzy repetitive control frameworks further strengthen frequency and current regulation in modern microgrid and inverter systems^17,18^.

To evaluate and compare the performance of the applied controllers, objective functions such as the integral of absolute error (IAE), integral of time-weighted absolute error (ITAE), integral of square error (ISE), and integral of time square error (ITSE) are employed^19^. Due to the practical challenges of experimental validation, hardware platforms such as OPAL-RT have been developed. These devices enable experimental verification of simulation results and are highly effective in modeling power systems^20^.

A more comprehensive literature review is presented in the next section.

### Literature review

In the literature, various aspects of multi-area systems—including the number and types of regions, the types of controllers used, the optimization algorithms applied for controller parameter tuning, and the objective functions employed—are summarized in Table 1.

**Table 1:** A brief literature survey of multi-area power systems, controller type, and optimization algorithms.

Year	Ref. no	Number of area/ energy unit	Type of energy production	Controller type	Optimization algorithm or method	Objective function
2024	^17^	5/10	Thermal Hydro/Thermal Wind/ Thermal Gas/ Hydro Diesel/ Hydro Wind	PID/FOFPID	mMSA	ITAE
2020	^18^	2/2	Non-reheat Thermal/Non-reheat Thermal	HODFC/FHODFC	PSO/COA/HSCOA	ITAE
		2/3	Re-Heat Thermal/Hydro/Gas			
2023	^19^	2/4	Thermal/Thermal	PI/PID	GA/PSO/TLBO	ITAE
2023	^20^	2/4	Non-reheat Steam Power/PV/ Non-reheat Steam Power/Wind	PI-PDN	GWO/PSO/BOA	ITAE
2024	^21^	2/2	Hydrothermal Power System/Hydrothermal Power System	TIDDF	CSOA	ISE
2024	^22^	5/10	Hydro/Wind/ Hydro/Diesel/ Diesel/Wind/ Hydro/Thermal/ Diesel/Thermal	FOPID-PR	AHB	ITAE
2023	^23^	2/2	Thermal/Thermal	2DOFPID	hGWO-PS	ITAE
2020	^24^	2/2	Hydro/Hydro	TDAC	Deep Reinforcement Learning Based TDAC	User Defined
2023	^25^	3/14	Multi Energy Production	Gaussian Interval-Based Type-2 Fuzzy PID Controller	Discrete Water Cycle Algorithm (DWCA)	ITAE
2024	^26^	2/6	Thermal/Hydro/Gas Thermal/Hydro/Gas	FOTID	SAR	ITAE
2023	^27^	2/2	Thermal/Thermal	ANFIS/ANN	PSO	ISE
2024	^28^	3/3	Thermal/Nuclear/Hydro	PID	GA/PSO/ACO	ITAE
2024	^29^	3/3	Thermal/Thermal/Thermal	DOF-PDN/ 2DOF-PDN/3DOF-PDN/ Neuro Fuzzy-3DOF-PDN	SOA	ISE
2023	^30^	2	Wind/PV	PID/FOPID	Coot Optimization Algorithm	ISE
2023	^31^	3/3	Reheat/Hydro/Gas	PI-PD	---	ISE/IAE/ITAE
2023	^32^	3/3	Non-Reheated Thermal/Non-Reheated Thermal	PID/FOPIDF/2DOFPID	MFO/SCA/ALO/SSA	ITAE
2024	^33^	3/6	Solar/Thermal Ocean/Termal & Nuclear Wind/Hydro	PID/Fuzzy 3DOFPID	BB-BC/LMA/FSO/PSO	ITSE
2021	^34^	3/3	Thermal/Thermal/Thermal + Flexible AC Transmission system	PDF + (1 + PI)	Grasshopper Optimization Algorithm	ITAE
2021	^35^	2/Multi-Microgrid	Thermal/Diesel Engine Generator/Micro Turbine/FC Fuel Cell/Photovoltaic/Wind Turbine/Battery/Flywheel Energy Storage System	Cascade PDF (1 + PI)	Imperialist Competitive Algorithm	ITAE
2022	^36^	1/Multi-DG (Microgrid)	PV/Wind/Diesel + EV/Battery	FO-T2F-PID	Improved Moth Swarm Algorithm	ISE
2021	^37^	2/4	Photovoltaic/Wind Turbine/HT Hydrothermal Unit/Hydrogen Aqua Equalizer–Fuel Cell	Fractional-Order Fuzzy PID (FOFPID)	Sunflower Optimization	ITAE
2024	^38^	1/Multi-DG (Microgrid)	Solar/Wind/Diesel/Fuel Cell + Energy Storage Devices	F-TIDF-2	Improved Equilibrium Optimization	ISE

As seen in Table 1, various controllers have been employed in multi-area power systems, including conventional PID^21–23^, PI-PD^24^, high-order differential feedback controller (HODFC) and fractional high-order differential feedback controller (FHODFC)^25^, series-connected controllers such as PI-PDN^26^, tilt-integral-double derivative filter (TIDDF)^27^, fractional order proportional integral derivative (FOPID)^28^, fractional order proportional integral derivative-proportional resonant (FOPID-PR)^29^, and 2DOFPID^30^; three-network double-delay actor-critic (TDAC)^31^, FLC of type 1 and 2, as well as PID-tuned FLC^32^, fractional order tilt-integral-derivative (FOTID)^33^; artificial neuro-fuzzy inference systems (ANFIS) and artificial neural networks (ANN)^34^; DOF-PDN, 2DOF-PDN, 3DOF-PDN, Neuro-Fuzzy 3DOF-PDN^23^, and 3DOFPID-Fuzzy 3DOFPID^35^.

Regarding optimization algorithms, various techniques have been employed, including modified moth swarm algorithm (mMSA)^21^, GA, teaching learning-based optimization (TLBO)^22^, particle swarm optimization (PSO)^25,26,34–36^, crow search optimization algorithm (CSOA)^27^, cuckoo optimization algorithm (COA) and COA integrated with the harmony search algorithm (HSCOA)^25^, bear optimization algorithm (BOA), GWO^26^, artificial hummingbird algorithm (AHB)^29^, hybrid grey wolf optimization-pattern search (hGWO-PS)^30^, deep reinforcement learning-based optimization and DWCA^32^, search and rescue (SAR) Algorithm^33^, ant colony optimization (ACO)^36^, skill optimization algorithm (SOA)^23^, coot optimization algorithm^28^, moth flame optimizer (MFO), sine–cosine algorithm (SCA), salp swarm algorithm (SSA), PSO, ant-lion optimization algorithm (ALO)^37^, big bang-big crunch (BB-BC) optimization, firebug swarm optimization (FSO), levenberg-marquardt algorithm (LMA), and PSO^35^. Additionally, numerous other optimization techniques have been explored in the literature.

The number of regions in the studies reviewed varies between two and five, with energy sources including thermal, photovoltaic, wind, hydro, and nuclear power. The number of energy generators in each region also differs depending on the system configuration.

Various controllers have been employed to regulate these systems. While PID-based controllers, different combinations of FOPID controllers, FLC, and DOFPID-based controllers are widely used, hybrid controllers such as Fuzzy-PID have also been commonly applied in load frequency control of multi-area power systems.

Finally, objective functions such as ITAE, IAE, ISE, and ITSE are frequently used for performance evaluation^21–42^. Among these, ITAE has been the most widely applied.

### Motivation

The literature review indicates that various controllers and algorithms have been tested in different multi-area power system configurations, yet no definitive conclusion has been reached. Structural differences between systems lead to variations in controller parameters, making it necessary to design controllers specific to each system. This raises important questions regarding which controller should be selected and how its parameters should be determined.

Based on previous studies, it was considered important to choose a simple yet effective controller to achieve better results. This approach was expected to provide advantages in terms of settling time and maximum overshoot. Additionally, relatively new and less commonly used optimization algorithms were explored for parameter tuning. Finally, the aim was to validate the simulation results experimentally using the OPAL-RT OP5707 device.

Although numerous studies have applied various optimization algorithms and controllers for multi-area power systems, several limitations can still be observed. First, most existing research has primarily focused on conventional optimization algorithms such as PSO, GA, and GWO, while relatively few studies have explored newly emerging metaheuristic methods with enhanced convergence capability. Second, many studies concentrated only on simulation-based validation without real-time experimental verification, limiting their practical applicability. Third, most previous models considered systems with two homogeneous generation units, whereas highly renewable and hybrid configurations combining solar, wind, and thermal sources remain insufficiently explored.

These limitations motivate the present study, which addresses all of the above issues by (i) employing a novel optimization algorithm (FATA) with balanced exploration and exploitation mechanisms, (ii) validating the results using an OPAL-RT real-time simulator, and (iii) considering a hybrid two-area system that integrates solar, wind, and thermal energy sources.

### Contribution

The main contributions and novelties of this study are summarized as follows:

- A two-area highly renewable hybrid power system, integrating solar, wind, and thermal generation units, was considered and analyzed for the LFC problem.
- The study introduces a novel application of the FATA for optimizing AGC parameters. To the best of our knowledge, this is the first implementation of FATA validated through a real-time simulation load frequency control using the OPAL-RT platform.
- A comprehensive comparative framework was designed, combining four controller types (PI, PIDn, FOPI, and PPIDn) with four optimization algorithms (GJO, ECO, ESC, and FATA) to identify the most effective configuration for frequency stabilization.
- The proposed approach was experimentally validated on the OPAL-RT OP5707 real-time simulator, demonstrating its practical feasibility and robustness under realistic operating conditions.

### Article organization

This article consists of five sections. The first section includes the Introduction, Literature Review, and Motivation, followed by the Article Organization. In the second section, information is provided on multi-area power systems, the controllers used for their regulation, and the applied optimization algorithms. The third section presents simulation studies and experimental results. The fourth section analyzes the obtained results, while the final section provides a general evaluation of the study and its findings. Additionally, recommendations are offered to guide future research.

**Fig. 2:**
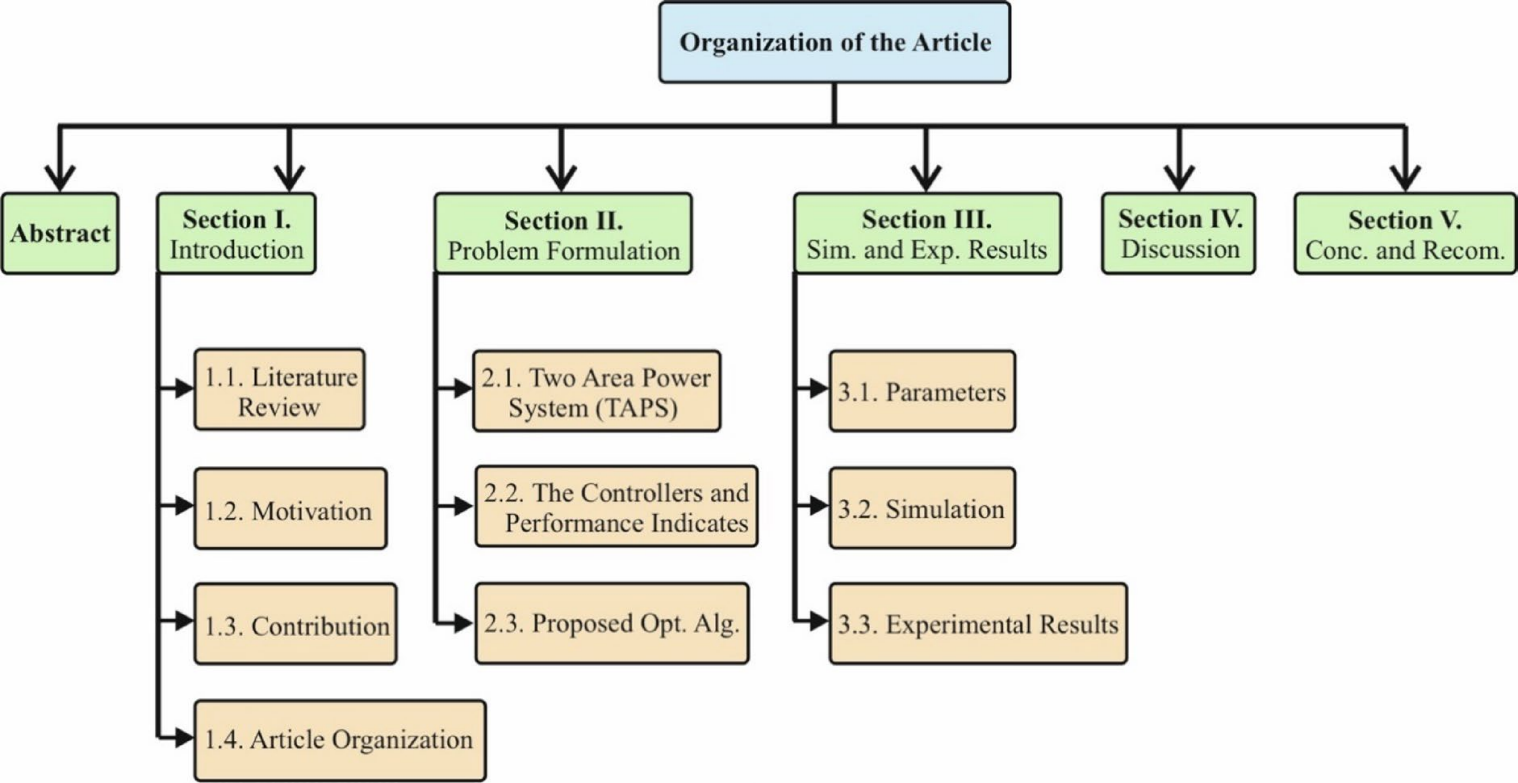
Organization of the paper.

Figure 2 illustrates the organizational structure of the article.

## Problem formulation

**Fig. 3:**
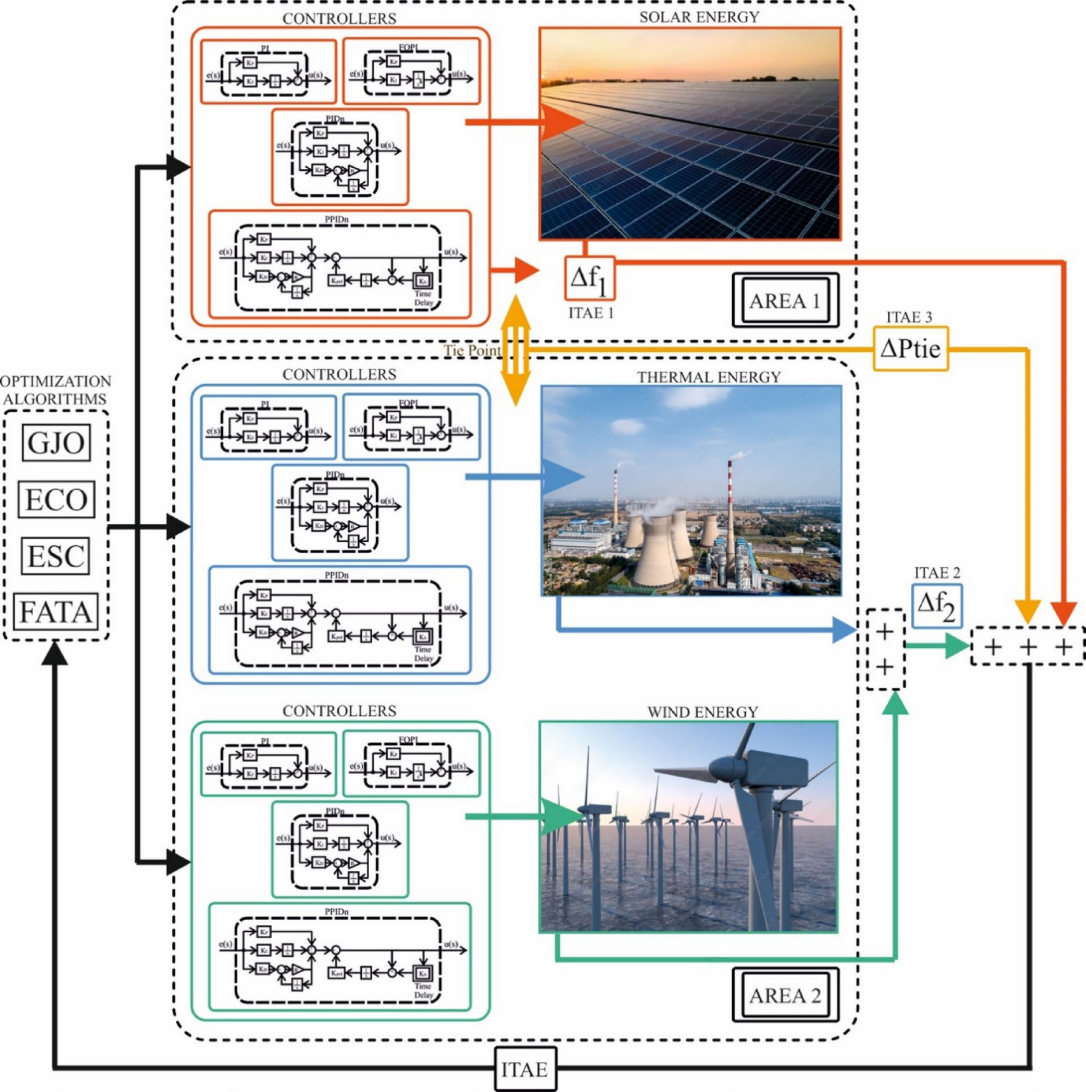
Two-area three power generation units power system and controller optimization.

Figure 3 illustrates a two-area power system incorporating three different energy generation sources: solar, thermal, and wind. The system has been controlled using four different controllers (PI, PIDn, FOPI, and PPIDn). To optimize the parameters of each controller, four optimization algorithms (GJO, ECO, ESC, and FATA) were applied. The objective was to determine the most effective combination of controller and optimization algorithm for achieving optimal system performance.

The optimization algorithms adjusted the controller parameters based on the ITAE criterion, which is widely used in the literature for evaluating control performance. By minimizing ITAE, the controllers were fine-tuned to enhance system stability and dynamic response. The overall structure of the system, along with the interaction between controllers, energy sources, and optimization methods, is schematically presented in Fig. 3.

### Two-area power system (TAPS)

In a two-area power system, the power transmitted through the transmission line is given by Eq. (1).

1 $$\:{P}_{line12}=\frac{\left|{V}_{1}\right|.\left|{V}_{2}\right|}{{X}_{12}}\text{sin}\left({\delta\:}_{1}-{\delta\:}_{2}\right)$$

In this equation, *V*_*1*_​ (V) and *V*_*2*_ (V) represent the voltages of area-1 and area-2, respectively, while *δ*_*1*_​ (^0^) and *δ*_*2*_​ (^0^) correspond to their phase angles. The term $\:{P}_{line12}$ denotes the power flow through the transmission line, and $\:{X}_{12}$​ represents the line impedance.

The rate of change of the phase angle in each area is given by Eq. (2), where $\:f$ (Hz) represents the system frequency.

2 $$\:\varDelta\:\delta\:=2\pi\:\int\:\varDelta\:fdt$$

3 $$\:{\varDelta\:P}_{12}=\frac{\left|{V}_{1}\right|.\left|{V}_{2}\right|}{{X}_{12}}\text{cos}\left({\delta\:}_{1}-{\delta\:}_{2}\right)\left(\varDelta\:{\delta\:}_{1}-{\varDelta\:\delta\:}_{2}\right)={T}_{12}\left(\varDelta\:{\delta\:}_{1}-{\varDelta\:\delta\:}_{2}\right)$$

4 $$\:{T}_{12}=\frac{\left|{V}_{1}\right|.\left|{V}_{2}\right|}{{X}_{12}}\text{cos}\left({\delta\:}_{1}-{\delta\:}_{2}\right)$$

5 $$\:{\varDelta\:P}_{tie}={T}_{12}\left(\varDelta\:{\delta\:}_{1}-{\varDelta\:\delta\:}_{2}\right)$$

The exchange of power between the regions is represented by Eq. (3), while the synchronizing torque coefficient is formulated in Eq. (4). The variation in power flow through the transmission line is expressed in Eq. (5). Instantaneous load fluctuations (*ΔP*_*L*_) lead to frequency deviations in the system. Consequently, the power variation and the Area Control Error (ACE) are mathematically defined in Eqs. (6) and (7), where $\:B$ denotes the frequency bias factor^1,3^.

6 $$\:{ACE}_{1}=-B\varDelta\:{f}_{1}-{\varDelta\:P}_{tie}$$

7 $$\:{ACE}_{2}=-B\varDelta\:{f}_{2}+{\varDelta\:P}_{tie}$$

The obtained values represent the error signals at the input of the controllers.

The TAPS system consists of three different energy generation units: a solar power system composed of photovoltaic (PV) panels, a thermal power generation station, and wind energy produced by wind turbines. The photovoltaic solar system constitutes area-1, while the thermal power station and wind turbines together form area-2.

**Fig. 4:**
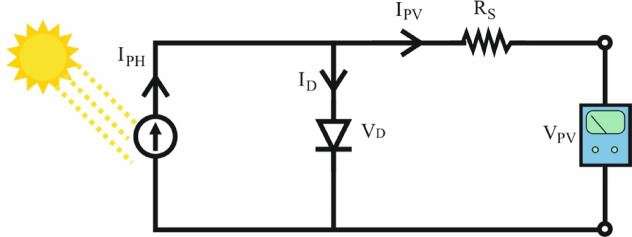
Solar cell equivalent circuit.

In area-1, photovoltaic solar panels are semiconductor devices that convert sunlight into electrical energy. Figure 4 illustrates a simplified equivalent circuit of a solar cell. Equation (8) expresses the voltage generated by a solar cell. By connecting multiple solar cells in series and parallel, solar panels are formed^43,44^.

8 $$\:{V}_{PV}=\frac{N}{\lambda\:}ln\left(\frac{{I}_{SC}-{I}_{PV}+M{I}_{0}}{M{I}_{0}}\right)-\frac{N}{M}{R}_{s}{I}_{PV}$$

The definitions and units of the PV variables are presented in Table 2.

**Table 2:** The descriptions for PV cell.

Expression		Definition	Unit
*N*	:	Series cells per string	
*λ*	:	Constant coefficient and depends upon the cell material	
*V*_*PV*_	:	Cell output voltage	V
*I*_*SC*_	:	Cell short circuit current	A
*I*_*PV*_	:	Cell output current	A
*I*_*PH*_	:	Photocurrent	A
*M*	:	Parallel strings	
*I*_*0*_	:	Reverse saturation current	A
*R*_*S*_	:	Series resistance of cell	Ω

The transfer function of the power generation system composed of solar panels is given in Eq. (9). This transfer function represents the overall dynamics of the inverter, filters, and maximum power point tracking (MPPT) system.

9 $$\:\text{P}\text{V}\:\text{S}\text{y}\text{s}\text{t}\text{e}\text{m}:\:{G}_{PV}\left(s\right)=\frac{a+bs}{{s}^{2}+cs+d}$$

where *a* corresponds to the negative value of the system’s zero, *c* and *d* represent the negative values of the poles, and *b* denotes the gain of the PV system^45,46^.

In area-2, two different power generation units are present: a thermal power plant and a wind energy system. The transfer functions of the thermal power system components, including the governor, reheat system, turbine, and power system, are expressed in Equations (10) to (13).

10 $$\:\text{G}\text{o}\text{v}\text{e}\text{r}\text{n}\text{o}\text{r}:\:{G}_{gov}\left(s\right)=\frac{{K}_{gov}}{s{T}_{gov}+1}$$

11 $$\:\text{T}\text{u}\text{r}\text{b}\text{i}\text{n}\text{e}:\:{G}_{tur}\left(s\right)=\frac{{K}_{tur}}{s{T}_{tur}+1}$$

12 $$\:\text{R}\text{e}\text{h}\text{e}\text{a}\text{t}\text{e}\text{r}:\:{G}_{reh}\left(s\right)=\frac{s{K}_{reh}{T}_{reh}+1}{s{T}_{p}+1}$$

13 $$\:\text{P}\text{o}\text{w}\text{e}\text{r}\:\text{S}\text{y}\text{s}\text{t}\text{e}\text{m}:\:{G}_{PS}\left(s\right)=\frac{{K}_{PS}}{s{T}_{PS}+1}$$

Table 3 provides definitions for the parameters used in the transfer functions of the thermal power system components, including the governor, reheater, turbine, and power system.

**Table 3:** The descriptions for thermal energy production unit.

Expression		Definition	Unit
*K*_*gov*_	:	Governor gain	p.u.
*K*_*tur*_	:	Turbine gain	p.u.
*K*_*reh*_	:	Reheater gain	p.u.
*K*_*ps*_	:	Power system gain	p.u.
*T*_*gov*_	:	Governor time constant	s
*T*_*tur*_	:	Turbine time constant	s
*T*_*reh*_	:	Reheater time constant	s
*T*_*ps*_	:	Power system time constant	s

The second power generation system in area-2 is the wind energy system. Wind turbines harness the kinetic energy of the wind to rotate their blades. The rotor is connected to a shaft that drives a generator operating in motor-generator mode, converting mechanical energy into electrical power^47^.

The mathematical representation of the output power generated by a wind turbine is given in Equations (14) to (16).

14 $$\:{P}_{wt}=\frac{1}{2}\rho\:{a}_{s}^{2}{V}^{3}{C}_{p}\left(TSR,\beta\:\right)$$

15 $$\:{C}_{p}=\left(TSR-0.022{\beta\:}^{2}-5.6\right){e}^{-0.17TSR}$$

16 $$\:TSR=\frac{{w}_{r}.\pi\:D}{60V}$$

The definitions of the terms used in the equations are provided in Table 4.

**Table 4:** Wind energy parameters.

Expression		Definition	Unit
*P*_*wt*_	:	Wind turbine mechanical output power	W
*ρ*	:	Air density	Kg/m^3^
*a*_*s*_	:	Swept area	m^2^
*V*	:	Wind speed	m/s
*C*_*p*_	:	Rotor efficiency	
*TSR*	:	Tip speed ratio	
*β*	:	Pitch angle of the blade	Degree
*ω*_*r*_	:	Rotor speed	rad/s
*D*	:	Blade rotor diameter	m

Based on these fundamental equations, the transfer functions of the wind turbine components have been derived and are presented in Equations (17) to (21)^48^.

17 $$\:\text{P}\text{i}\text{t}\text{c}\text{h}\:\text{C}\text{o}\text{n}\text{t}\text{r}\text{o}\text{l}:\:{G}_{pc}\left(s\right)=\frac{{K}_{p1}(s{T}_{p1}+1)}{s+1}$$

18 $$\:\text{H}\text{y}\text{d}\text{r}\text{a}\text{u}\text{l}\text{i}\text{c}\:\text{P}\text{i}\text{t}\text{c}\text{h}\:\text{A}\text{c}\text{t}\text{u}\text{a}\text{t}\text{o}\text{r}:\:{G}_{hpa}\left(s\right)=\frac{{K}_{p2}}{s{T}_{p2}+1}$$

19 $$\:\text{D}\text{a}\text{t}\text{a}\:\text{F}\text{i}\text{t}\:\text{P}\text{i}\text{t}\text{c}\text{h}:\:{G}_{dfp}\left(s\right)=\frac{{K}_{p3}}{s{T}_{p3}+1}$$

20 $$\:\text{I}\text{n}\text{d}\text{u}\text{c}\text{t}\text{i}\text{o}\text{n}\:\text{G}\text{e}\text{n}\text{e}\text{r}\text{a}\text{t}\text{o}\text{r}:\:{G}_{IG}\left(s\right)=\frac{1}{s{T}_{w}+1}$$

21 $$\:\text{O}\text{u}\text{t}\text{p}\text{u}\text{t}\:\text{W}\text{i}\text{n}\text{d}\:\text{P}\text{o}\text{w}\text{e}\text{r}\:\text{D}\text{e}\text{v}\text{i}\text{a}\text{t}\text{i}\text{o}\text{n}:\:{\varDelta\:P}_{wt}\left(s\right)={K}_{fc}{G}_{IG}\left(s\right)$$

The descriptions of the equations used in Equations (17) to (21) are provided in Table 5.

**Table 5:** Descriptions and units of the variables used in the transfer functions.

Expression		Definition	Unit
*K*_*p1*_	:	Pitch control gain	p.u.
*K*_*p2*_	:	Hydraulic pitch actuator gain	p.u.
*K*_*p3*_	:	Data fit pitch gain	p.u.
*K*_*fc*_	:	Fluid coupling gain	p.u.
*T*_*p1*_	:	Pitch control time constant	s
*T*_*p2*_	:	Hydraulic pitch actuator time constant	s
*T*_*p3*_	:	Data fit pitch time constant	s
*T*_*w*_	:	Induction generator time constant	s

### The controllers and performance indices

**Fig. 5:**
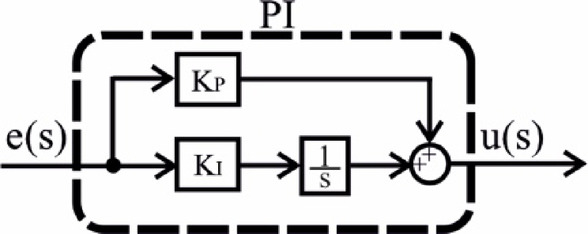
PI controller.

**Fig. 6:**
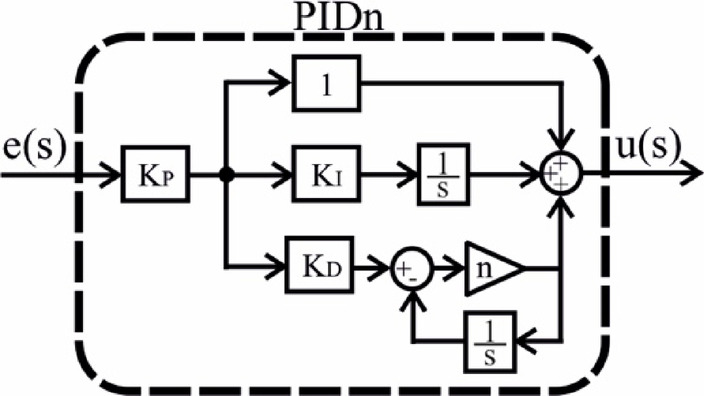
PIDn controller.

**Fig. 7:**
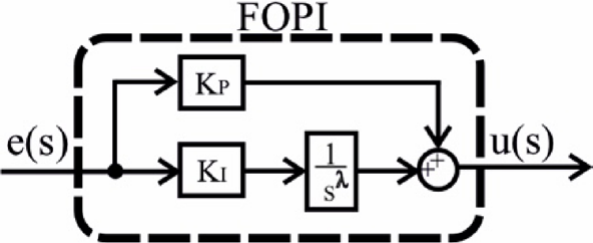
FOPI controller.

**Fig. 8:**
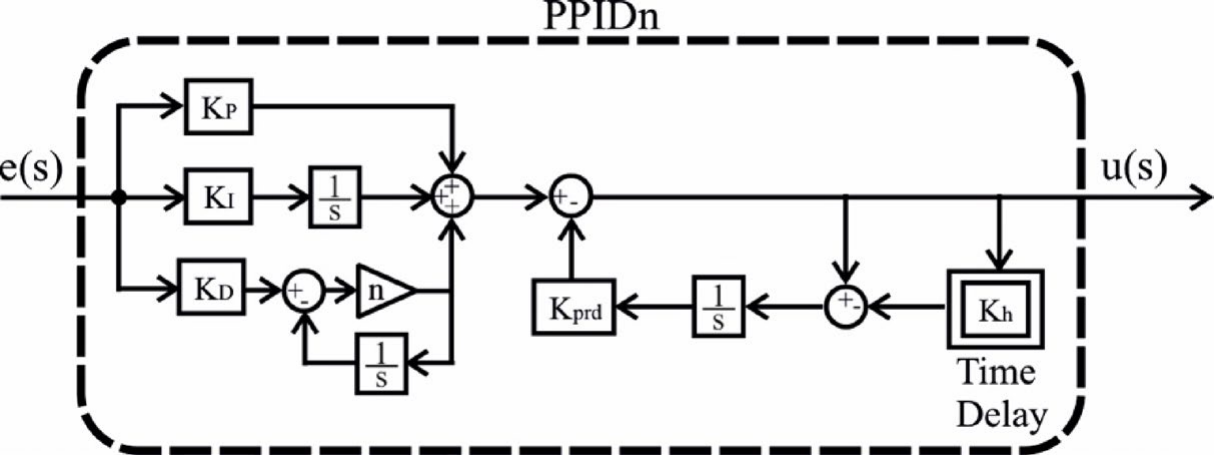
PPIDn controller.

In this study, four different controllers were selected and their parameters were optimized. The selection was based on a comprehensive literature review, focusing on controllers expected to exhibit high performance. As a result, PI, PIDn, FOPI, and PPIDn controllers were chosen. The structural representations of these controllers are illustrated in Figs. 5, 6, 7 and 8, while their mathematical formulations are provided in Equations (22) to (25) where the ideal connection forms of the PIDn and PPIDn controllers are performed.

22 $$\:{\frac{u\left(s\right)}{e\left(s\right)}=G}_{PID}={K}_{P}+\frac{{K}_{I}}{s}$$

23 $$\:{\frac{u\left(s\right)}{e\left(s\right)}=G}_{PIDn}={K}_{P}\left(1+\frac{{K}_{I}}{s}+{K}_{D}\left(\frac{ns}{s+n}\right)\right)$$

24 $$\:{\frac{u\left(s\right)}{e\left(s\right)}=G}_{FOPID}={K}_{P}+\frac{{K}_{I}}{{s}^{\lambda\:}}$$

25 $$\:U\left(s\right)=\left({K}_{p}+\frac{{K}_{I}}{s}+s{K}_{D}\frac{n}{s+n}\right)E\left(s\right)-\frac{{K}_{prd}}{s}\left(1-{e}^{-s{K}_{h}}\right)U\left(s\right)$$

The descriptions of the terms used in Equations (22) to (25) are provided in Table 6.

**Table 6:** The controller parameters.

Expression		Definition
*K*_*p*_	:	Proportional gain
*K*_*D*_	:	Derivative gain
*K*_*I*_	:	Integral gain
*K*_*prd*_	:	Predictive term constant
*K*_*h*_	:	Delay time of the predictive term
*n*	:	Derivative action filter constant
λ		Fractional integrator order

To compare the performance of the controllers, error performance indices, as defined in Equations (26) to (29), were utilized. This approach is consistent with findings from the literature review. Typically, one of these indices is selected to evaluate control performance. In this study, ITAE was chosen due to its widespread use.

26 $$\:\text{I}\text{S}\text{E}=\underset{0}{\overset{{\infty\:}}{\int\:}}{\text{e}}^{2}\left(\text{t}\right)\text{d}\text{t}$$

27 $$\:\text{I}\text{A}\text{E}=\underset{0}{\overset{{\infty\:}}{\int\:}}\left|\text{e}\left(\text{t}\right)\right|\text{d}\text{t}$$

28 $$\:\text{I}\text{T}\text{S}\text{E}=\underset{0}{\overset{{\infty\:}}{\int\:}}\text{t}{\text{e}}^{2}\left(\text{t}\right)\text{d}\text{t}$$

29 $$\:\text{I}\text{T}\text{A}\text{E}=\underset{0}{\overset{{\infty\:}}{\int\:}}\text{t}\left|\text{e}\left(\text{t}\right)\right|\text{d}\text{t}$$

Another reason to employ ITAE is that it provides a balanced trade-off between transient and steady-state responses. In LFC, long-term deviations are often more critical than short-term overshoots. Therefore, the time-weighting factor in ITAE effectively penalizes prolonged oscillations while ensuring faster settling with minimal steady-state error. This makes ITAE a more appropriate index for evaluating controller performance in interconnected power systems compared to other popular indices such as ISE, IAE, and ITSE.

In the simulation, the objective function, presented in Eq. (30), was derived from the sum of the errors in each region and the interconnection point. The optimization algorithms were then evaluated based on this criterion. (st: simulation time)

30 $$\:{OF}_{ITAE}=\underset{0}{\overset{\text{s}\text{t}}{\int\:}}\text{t}\left|{\varDelta\:}_{f1}\right|\text{d}\text{t}+\underset{0}{\overset{\text{s}\text{t}}{\int\:}}\text{t}\left|{\varDelta\:}_{f2}\right|\text{d}\text{t}+\underset{0}{\overset{\text{s}\text{t}}{\int\:}}\text{t}\left|{\varDelta\:}_{Ptie}\right|\text{d}\text{t}$$

In this equation, Eq. (29) was applied based on the frequency deviations in each region and at the interconnection point^49,50^.

### Proposed optimization algorithm

In this study, the parameters of four different controllers (PI, PIDn, FOPI, and PPIDn) were optimized using four optimization algorithms: GJO, ECO, ESC, and FATA. Each algorithm was selected based on its potential effectiveness in optimizing control parameters. A comparative analysis was conducted to evaluate their performance, with a particular emphasis on the FATA algorithm, which demonstrated superior results in this context.

#### Overview of the FATA

The Fata Morgana Algorithm (FATA) is a newly developed optimization technique inspired by the Fata Morgana optical phenomenon, a complex mirage effect caused by atmospheric refraction. This phenomenon creates illusions of floating landscapes or distorted images due to variations in air density and light refraction. By mimicking these principles, the FATA algorithm introduces a novel approach to balancing global exploration and local exploitation in optimization tasks.

Unlike conventional metaheuristic algorithms that rely on predefined search mechanisms, FATA dynamically adjusts its search behavior through two key principles:

1. The mirage light filtering principle, which selectively refines the population based on an integral-based evaluation method, enhancing solution quality.
2. The light propagation principle, which governs the movement and adaptation of solutions, ensuring efficient exploration and convergence.

By integrating these mechanisms, FATA provides a robust framework for solving complex optimization problems, effectively avoiding local optima while maintaining convergence efficiency. The following sections detail the inspiration behind FATA, its mathematical formulation, and its application in controller optimization.

#### Inspiration behind the Fata Morgana phenomenon

**Fig. 9:**
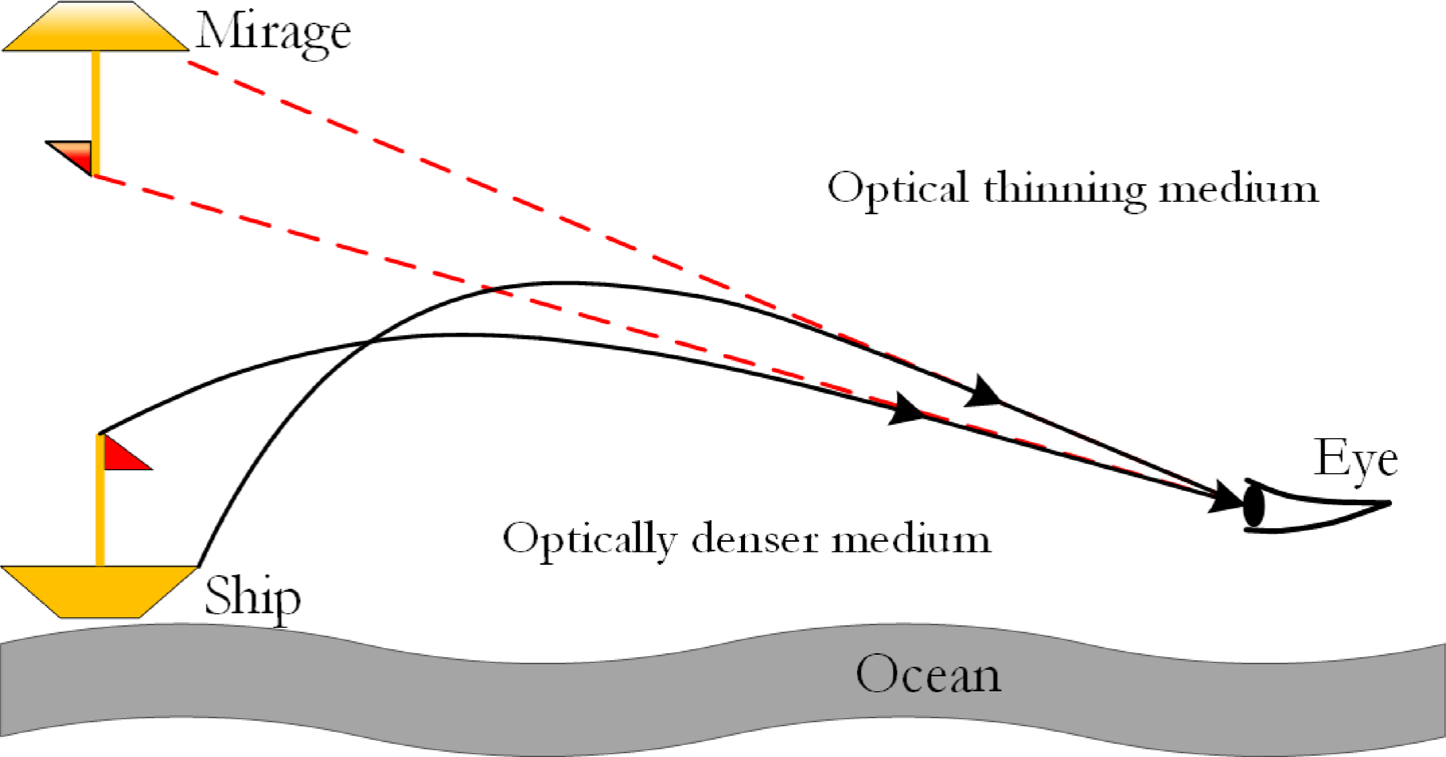
Mirage Formation Process^51^.

The fata morgana, or mirage, is a naturally occurring optical phenomenon. It arises due to the behavior of light as it propagates through an atmosphere with varying density, transitioning from an optically denser medium to a less dense one. This study investigates the formation of mirages by examining light rays emitted from underwater features. It also inspires the design depicted in Fig. 9. The figure demonstrates the optical path of light rays emitted from a ship at sea, which ultimately create a mirage. The formation of a mirage necessitates two conditions: a medium with non-uniform density and the propagation of light through this medium. Initially, solar heating induces temperature variations in the atmosphere, creating the required inhomogeneous density gradient. When light from the ship reflects into this medium, its refraction angle continuously changes during propagation, culminating in total internal reflection, which produces the mirage effect. An observer (as shown by the Eye in Fig. 9) perceives this phenomenon when looking towards the sky in a specific direction (red)^51^.

As illustrated in Fig. 9, the formation of a mirage depends on the delicate balance between filtering mirage light and managing light refraction and reflection during propagation. Similarly, a parallel can be drawn to swarm intelligence algorithms, where achieving a balance between global exploration and local exploitation remains a challenge. Current algorithms, such as the Harris Hawks Optimization (HHO), sequentially implement global and local search strategies, using soft and hard besiege mechanisms inspired by hawk hunting. However, these strategies often lack the fine balance observed in the mirage phenomenon.

The mirage principle, illustrated in Fig. 9, offers an innovative approach to algorithm design by harmonizing global and local search strategies. In this figure, light emitted by a ship enters an atmosphere with a non-uniform density. As it transitions from an optically denser medium to a less dense one, the refractive index changes, causing the light to bend at progressively larger angles. Upon reaching the critical angle, total internal reflection occurs, resulting in the formation of a mirage. This dynamic balance between refraction and reflection processes serves as the conceptual foundation for a new optimization framework.

Building upon this concept, the paper introduces two key principles essential to the proposed FATA: the mirage light filtering principle and the light propagation principle. The former governs the selection process, allowing specific light waves to contribute to the mirage while excluding others, whereas the latter dictates how light adjusts its direction and intensity as it travels through a medium with varying density. These principles play a crucial role in shaping the behavior of swarm intelligence algorithms.

The FATA algorithm derives its global search strategy (mirage light filtering principle) from the light reflected by the ship into the medium, while its local search strategy (light propagation principle) is inspired by the refraction and total internal reflection of light. Together, these principles form the core of the algorithm, achieving a balance between exploration and exploitation.

By emulating the mirage formation process, FATA integrates these strategies seamlessly, creating a robust optimization framework. This alignment between the optical phenomenon and the algorithm’s design ensures consistency and effectiveness, establishing the FATA as a novel and balanced optimization tool.

#### Fata Morgana algorithm

**Fig. 10:**
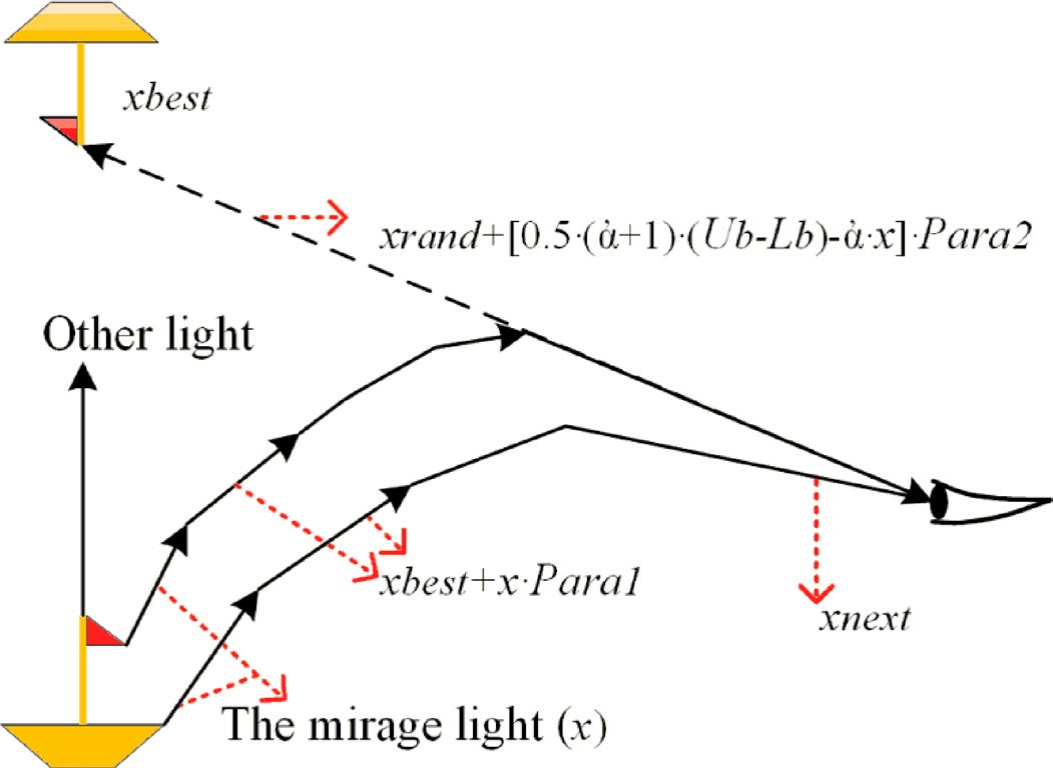
FATA optimization process in three dimensions^51^.

As depicted in Fig. 10, the FATA represents the population as multiple light rays that contribute to forming a mirage, with each light ray ($\:x$) representing an individual within the population. The mirage ($\:{x}_{best}$) serves as the optimization target.

At the initial stage, the population of light rays undergoes a dynamic assessment based on the mirage light filtering principle, which is rooted in the definite integral concept. This process evaluates the light emitted from the hull, specifically located in the lower-left corner of Fig. 10. Within this population, some light rays undergo physical transformations to form the mirage ($\:{x}_{best}$), while others, though similarly transformed, propagate in directions that do not contribute to forming the mirage.

In the second stage, the filtered light population undergoes the light propagation strategy, encompassing processes of refraction in the first and second stages and total internal reflection. The propagation of light through a medium with an inhomogeneous density mirrors the exchange of information between individuals in the algorithm. Through this process, the algorithm iteratively explores the solution space, leveraging physical changes in light propagation to identify the target mirage ($\:{x}_{best}$), which corresponds to the optimal solution^51^.

##### The Mirage light filtering principle

This section outlines the population search mechanism of FATA, drawing inspiration from the definite integral principle. As shown in Fig. 10, the hull emits two different types of light rays during the mirage formation process. The first type, labeled as ‘other light’ in Fig. 10, plays no role in forming the mirage and dissipates without undergoing notable changes. The second type, termed “mirage light ($\:x$),” undergoes physical changes, ultimately forming the mirage^51^.

**Fig. 11:**
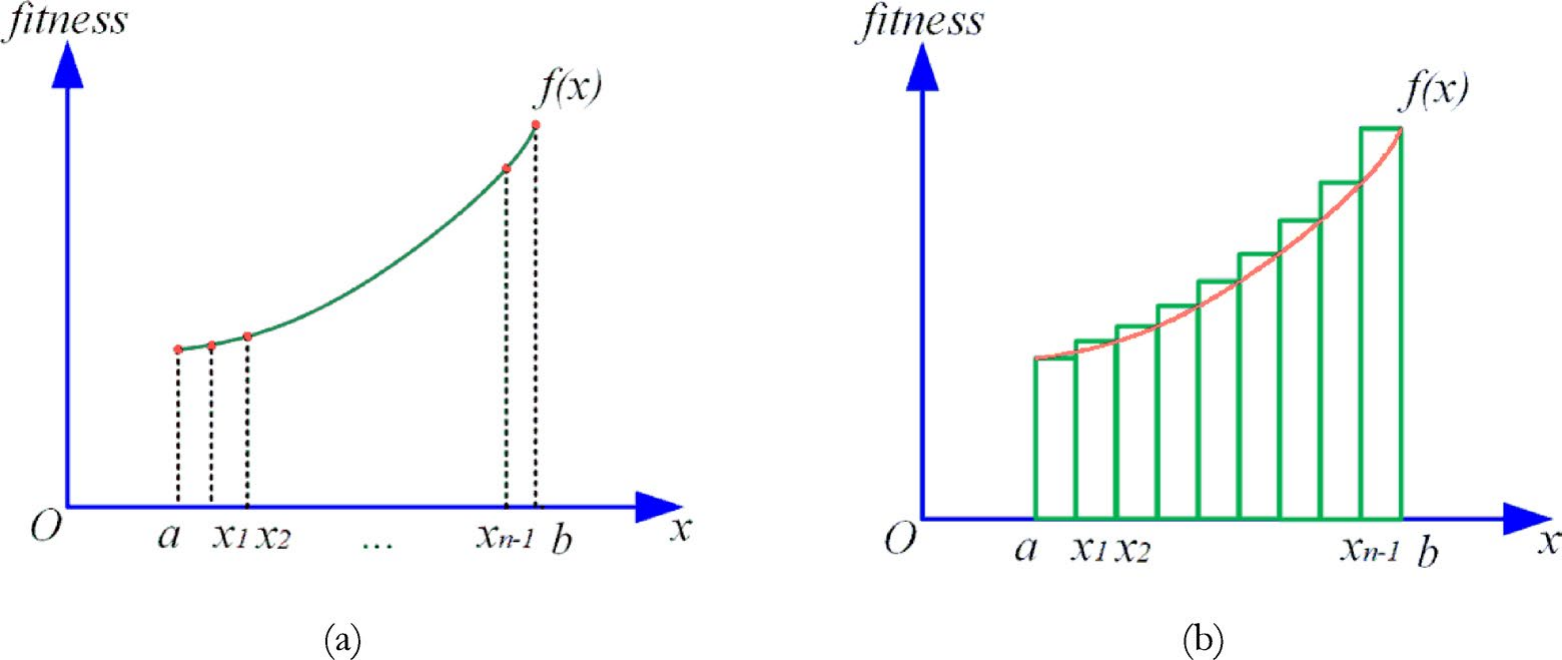
Population fitness curve in FATA (**a**) Population fitness function curve (**b**) Integrated area (S) Under the curve^51^.

In FATA, differentiating between these two light populations is essential to identifying the optimal solution, $\:{x}_{best}$​. To achieve this, FATA adopts a population quality evaluation mechanism grounded in the principle of definite integrals, enabling effective distinction and selection of relevant populations. Analogous to swarm intelligence algorithms, the quality of a population is measured by aggregating the fitness of individuals within it. As shown in Fig. 11a, ranking the fitness of individuals in a population creates a cumulative curve. To streamline the fitness computation of the two types of light populations (“other light” and “mirage light”), FATA applies definite integration to evaluate the area under this curve (Fig. 11b). The resulting integral value serves as an indicator of population fitness. Using this approach, FATA isolates the filtered mirage light population, which represents individuals selected based on their integral-based fitness evaluation.

This method enables FATA to efficiently balance exploration and exploitation by focusing on high-quality populations, ensuring robust optimization performance.

The FATA employs a fitness evaluation strategy to classify the population into two categories: “other light” and “mirage light,” based on population quality. The population quality refers to the overall performance or effectiveness of the population. In this strategy, the integrated area ($\:S$) under the population fitness function ($\:f\left(x\right)$) curve is used as a measure of population quality. Figure 11a illustrates the curve representing the population fitness function, while Fig. 11b depicts the integrated area ($\:S$) beneath this curve.

In swarm intelligence algorithms (SIAs), fitness typically represents the quality of individual solutions. However, evaluating the overall population quality becomes challenging when fitness values are discrete and high-dimensional. To address this, individual fitness values are approximated using a continuous function ($\:f\left(x\right)$). The FATA leverages the principle of definite integration to compute the integrated area ($\:S$) of the population fitness function curve. This integrated area serves as a comprehensive metric to assess and compare the quality of different populations.

By quantifying population quality through definite integration, FATA ensures an efficient and reliable mechanism for distinguishing high-quality populations, enabling the application of appropriate search strategies tailored to either the “other light” or “mirage light” populations^51^.

31 $$\:{x}_{i}^{next}=\left\{\begin{array}{c}{L}_{b}+\left({U}_{b}-{L}_{b}\right)\bullet\:rand\:\:\:\:\:\:\:\:\:\:\:\:\:\:\:\:\:\:\:\:\:\:\:\:\:\:\:\:\:\:\:\:\:\:\:\:\:\:\:\:\:\:\:\:\:\:\:\:\:\:\:\:\:\:\:\:\:\:\:\:\:\:\:\:\:\:\:\:\:\:\:\:\:\:\:\:\:\:\:,rand>P\\\:{x}_{best}+{x}_{i}\bullet\:{Para}_{1}\:\:\:\:\:\:\:\:\:\:\:\:\:\:\:\:\:\:\:\:\:\:\:\:\:\:\:\:\:\:\:\:\:\:\:\:\:\:\:\:\:\:\:\:\:\:\:\:\:\:\:\:\:\:\:\:\:\:,\:rand\le\:P\:\:and\:\:rand<q\\\:{x}_{rand}+[0.5\bullet\:\left(\alpha\:+1\right)\left({U}_{b}-{L}_{b}\right)-\alpha\:{x}_{i}]\bullet\:{Para}_{2}\:\:,\:rand\le\:P\:\:and\:\:rand\ge\:q\end{array}\right.$$

32 $$\:P=\frac{S-{S}_{worst}}{{S}_{best}-{S}_{worst}}$$

33 $$\:q=\frac{{fit}_{i}-{fit}_{worst}}{{fit}_{best}-{fit}_{worst}}$$

In the FATA, $\:x$ represents an individual light, while $\:{x}^{next}$ denotes the new individual generated during the optimization process. Algorithm 1 outlines the mirage light filtering principle employed by FATA. This filtering process includes three primary strategies: the first-half refraction strategy, the second-half refraction strategy, and the total internal reflection strategy, which are detailed sequentially in Sect. 3.2.

The population quality factor ($\:P$) is defined in Eq. (31), where a smaller value of the integrated area ($\:S$) indicates a higher-quality population. Specifically, $\:{S}_{worst}$​ and $\:{S}_{best}$​ represent the quality of the worst and best populations, respectively. Populations classified as mirage light populations exhibit superior quality, characterized by smaller $\:S$ values.

In Eq. (31), the individual quality factor ($\:q$) is introduced, which quantifies the performance of each individual within the population. Here, $\:{fit}_{i}$ ​ represents the fitness of the current individual ($\:x$), $\:{fit}_{worst}$ corresponds to the fitness of the worst-performing individual, and $\:{fit}_{best}$​ represents the fitness of the best individual. By leveraging these fitness metrics and quality factors, FATA effectively identifies and prioritizes high-performing mirage light populations and individuals to guide the optimization process^51^.

**Algorithm 1:**
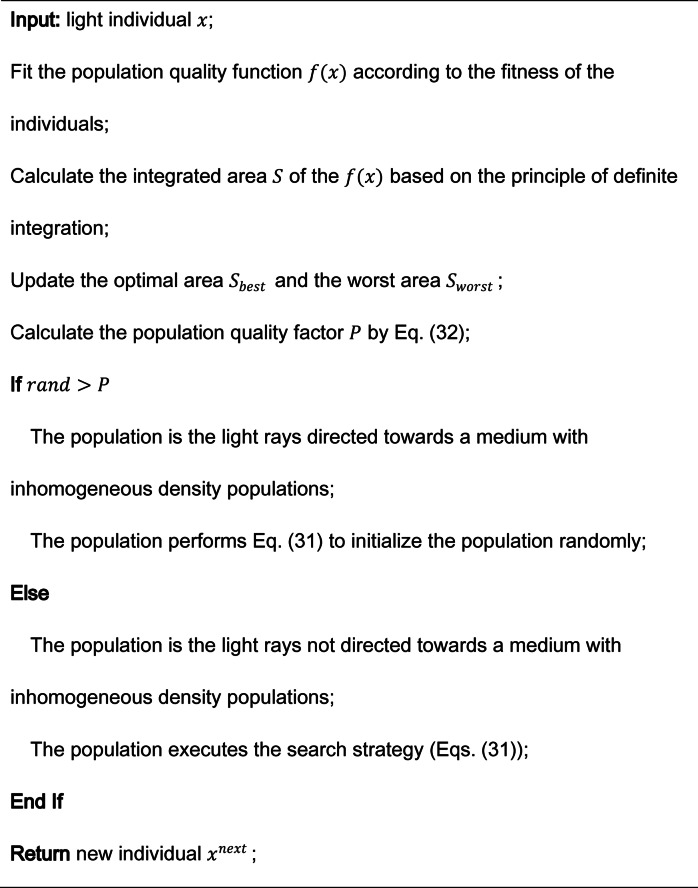
The mirage light filtering strategy

34 $$\:y=f\left(x\right)=\sum\:_{j=0}^{n}{c}_{j}{\phi\:}_{j}x$$

35 $$\:S={\int\:}_{a}^{b}f\left(x\right)dx\approx\:\frac{b-a}{n}\bullet\:(\frac{{y}_{0}+{y}_{1}}{2}+\frac{{y}_{1}+{y}_{2}}{2}+\dots\:+\frac{{y}_{n-1}+{y}_{n}}{2})$$

Equations (34–35) detail the process of calculating the area ($\:S$) under the population fitness curve $\:f\left(x\right)$ ($\:f\left({x}_{1}\right)<f\left({x}_{2}\right)\dots\:<f\left({x}_{i}\right)\dots\:<f\left({x}_{n}\right)$) using the principle of definite integration. This principle leverages the concept of limits to compute the integrated area of $\:f\left(x\right)$. Specifically, Eq. (34) defines the population quality fitting function $\:f\left(x\right)$, which represents the fitness curve using discrete points $\:({x}_{i},{y}_{i})$, where $\:i\in\:[1,n]$. The parameters $\:{c}_{j}$ and $\:{\phi\:}_{j}$ are used within the fitting function to optimize the representation of the curve. This approach ensures accurate estimation of population quality by evaluating the overall fitness distribution within the population.

##### Light propagation principle

**Fig. 12:**
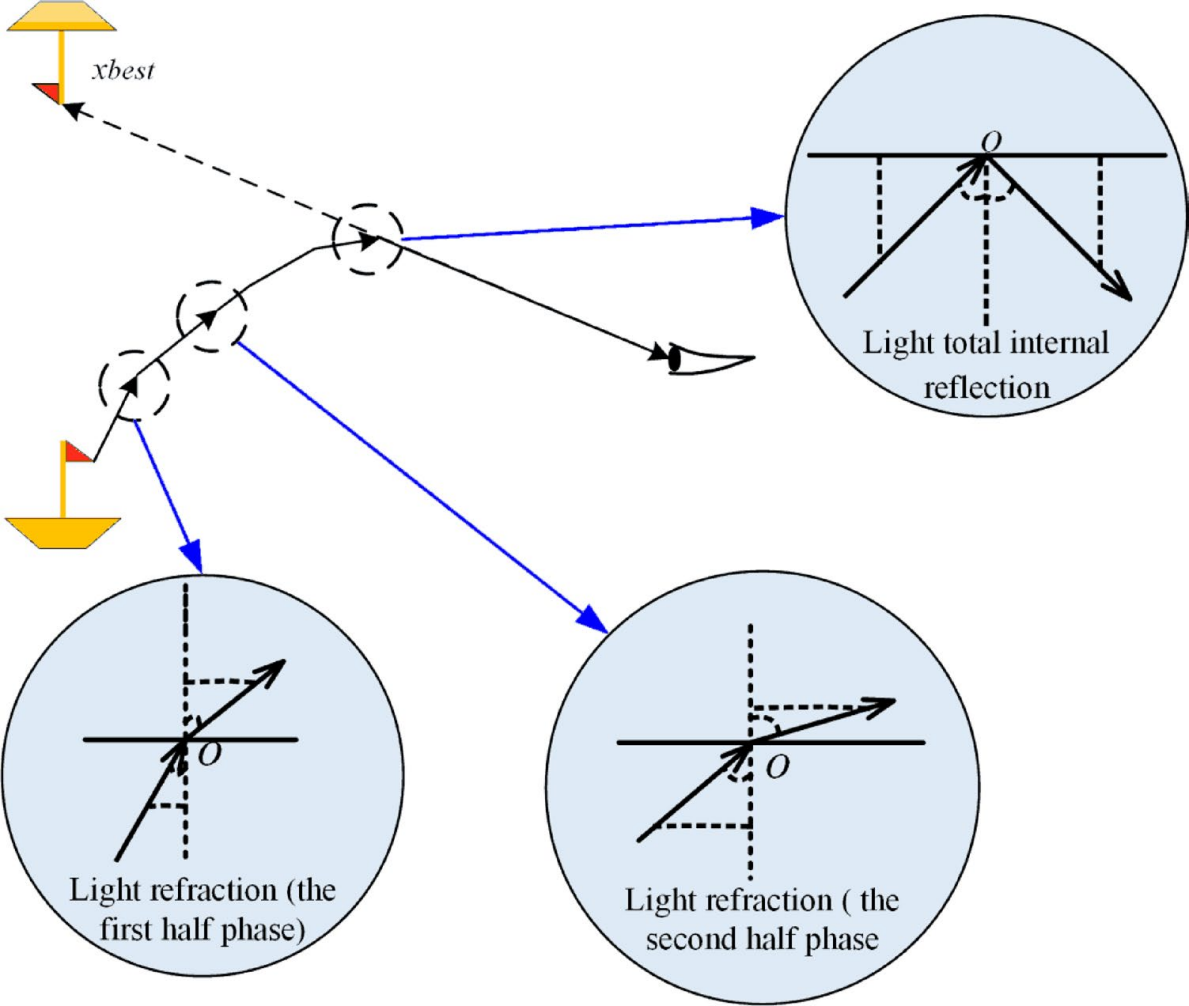
FATA algorithm and the mirage principle^51^.

In FATA, the light propagation principle is applied following the mirage light filtering strategy. This mechanism functions as the algorithm’s local search approach, enhancing exploitation within the search space to identify minima. As shown in Fig. 12, the mirage light rays in FATA originate from a small boat positioned in the lower-left corner of the search space.

Initially, the light population undergoes the mirage light filtering process, where the population is evaluated and refined using calculus-based principles to identify individuals contributing to the mirage phenomenon. After this filtering, the refined mirage light population is subjected to sequential refraction and reflection processes. During these stages, the direction and size of the light rays adapt dynamically, as depicted in Fig. 12, showcasing their transformations. This iterative procedure allows the filtered light population to explore the local search space effectively, optimizing the chances of locating a local minimum.

FATA employs a distinct search strategy based on the light propagation principle, further refined using trigonometric functions. This approach consists of three sequential components: the refraction strategy in the initial phase, the reflection strategy in the subsequent phase, and a final refraction step. The application of these strategies is governed by the individual quality factor defined in Eq. (35).

**Fig. 13:**
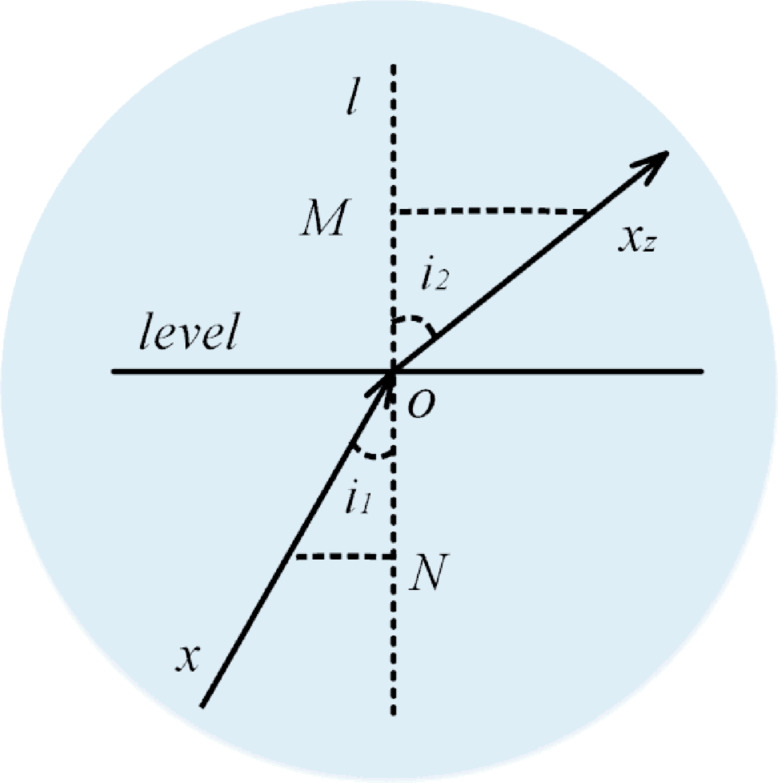
First refraction process of light.

Light Refraction (First Half Phase): In this phase, illustrated in Figure 13, light rays (denoted as $x$) enter a medium with varying density, transitioning from an optically denser medium to a less dense medium. During this transition, both the direction and size of the light rays are altered. The relationship between the angle of incidence (​$i_{1}$) and the angle of refraction ($i_{2}$) follows the principle that ​$i_{1} < i_{2}$.

Figure 13 visually analyzes the process of refraction for the light individuals. The light ray is represented as $\:x$, and the refractive surface is labeled as the level. After undergoing the first half of the refraction process, a new individual,), $\:{x}^{next}$, is generated, as defined by Eq. (36). The relationship $\:NO=C\bullet\:OM$, where $\:C$ is a constant, is assumed for this phase. The equations (Eqs. 36–38) govern the transformations in this strategy, guiding the propagation of light rays through the medium.

36 $$\:x^{{next}} = x_{{best}} + x_{z}$$

37 $$\:x_{z} = x \bullet \:Para_{1}$$

38 $$\:Para_{1} = \frac{{\sin \left( {i_{1} } \right)}}{{C \bullet \:\cos \left( {i_{2} } \right)}} = \tan \left( {\theta \:} \right)$$

In the FATA, the propagation of light individuals involves dynamic adjustments during the first-half refraction strategy. Here, $\:{x}^{next}$ denotes the newly generated individual, while $\:{x}_{best}$​ represents the current best individual. The refraction step is symbolized as $\:{x}_{z}$​, which describes the intermediate adjustment of the individual during the refraction process.

A key parameter in this strategy is $\:{Para}_{1}$​, the first-half refraction ratio, which dynamically changes during light propagation. This parameter regulates the degree of transformation the light individual undergoes when passing from one medium to another.

To simplify the measurement of the incident angle ($\:{i}_{1}$​) and the refraction angle ($\:{i}_{2}$​), the algorithm introduces the parameter $\:\theta\:$, which substitutes the angle variation. In the algorithm, $\:\theta\:$ is defined within the range $\:\left[\text{0,1}\right]$, providing a normalized and efficient means to represent the angle change during the refraction process. Equation (38) encapsulates this relationship, ensuring accurate computation of the transformations during light propagation^51^.

**Fig. 14:**
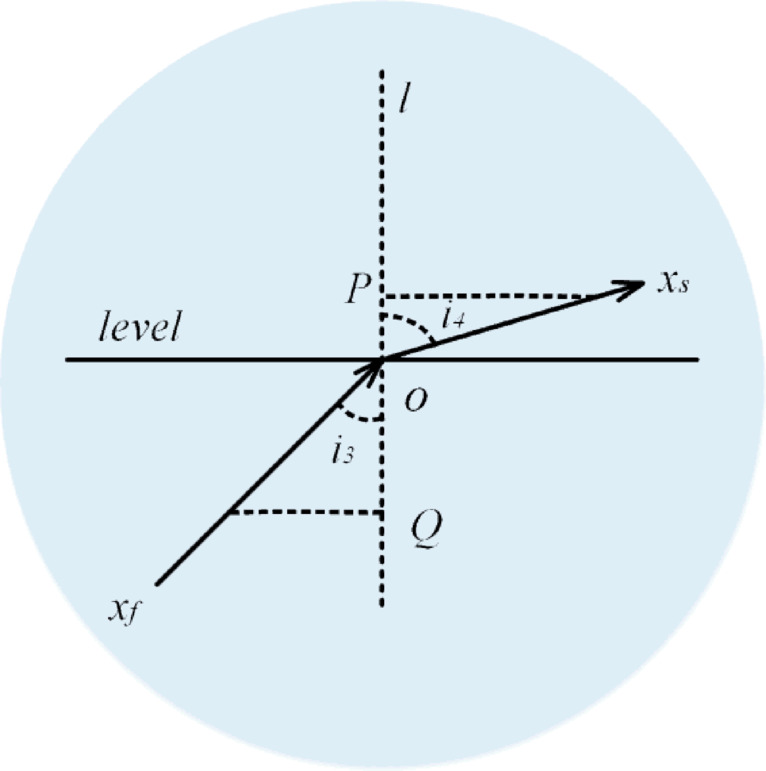
Second refraction process of light.

Light Refraction (Second Half Phase): Following the first half refraction phase, the light enters the second half refraction phase, where it propagates through random points within the medium. As illustrated in Fig. 14, the process is characterized by the angle of incidence ($i_{3}$) being smaller than the angle of refraction ($i_{4}$). This occurs because the light travels through a medium with varying density, resulting in a continuously changing refractive index ($Para_{2}$ ​).

During this phase, the light individual ($\:{x}_{f}$​) generates a new individual ($\:{x}^{next}$) by incorporating information from random individuals ($\:{x}_{rand}$​) located within the search space. This random selection enhances the exploration capabilities of the algorithm. The transformations involved in this refraction process are mathematically described in Eqs. (41–43), which outline the systematic generation of new individuals in the context of FATA’s refraction strategy.

39 $$\:x^{{next}} = x_{{rand}} + x_{s}$$

40 $$\:{x}_{s}={x}_{f}\bullet\:{Para}_{2}$$

41 $$\:{Para}_{2}=\frac{{cos}\left({i}_{5}\right)}{C\bullet\:sin\left({i}_{6}\right)}=\frac{1}{tan\left(\theta\:\right)}$$

In the second half refraction strategy, $\:{x}_{s}$​ represents the refraction step, while $\:{x}_{rand}$​ denotes a randomly selected individual from the population. The second refraction ratio, $\:{Para}_{2}$​, plays a key role in determining the behavior of light propagation during this phase.

**Fig. 15:**
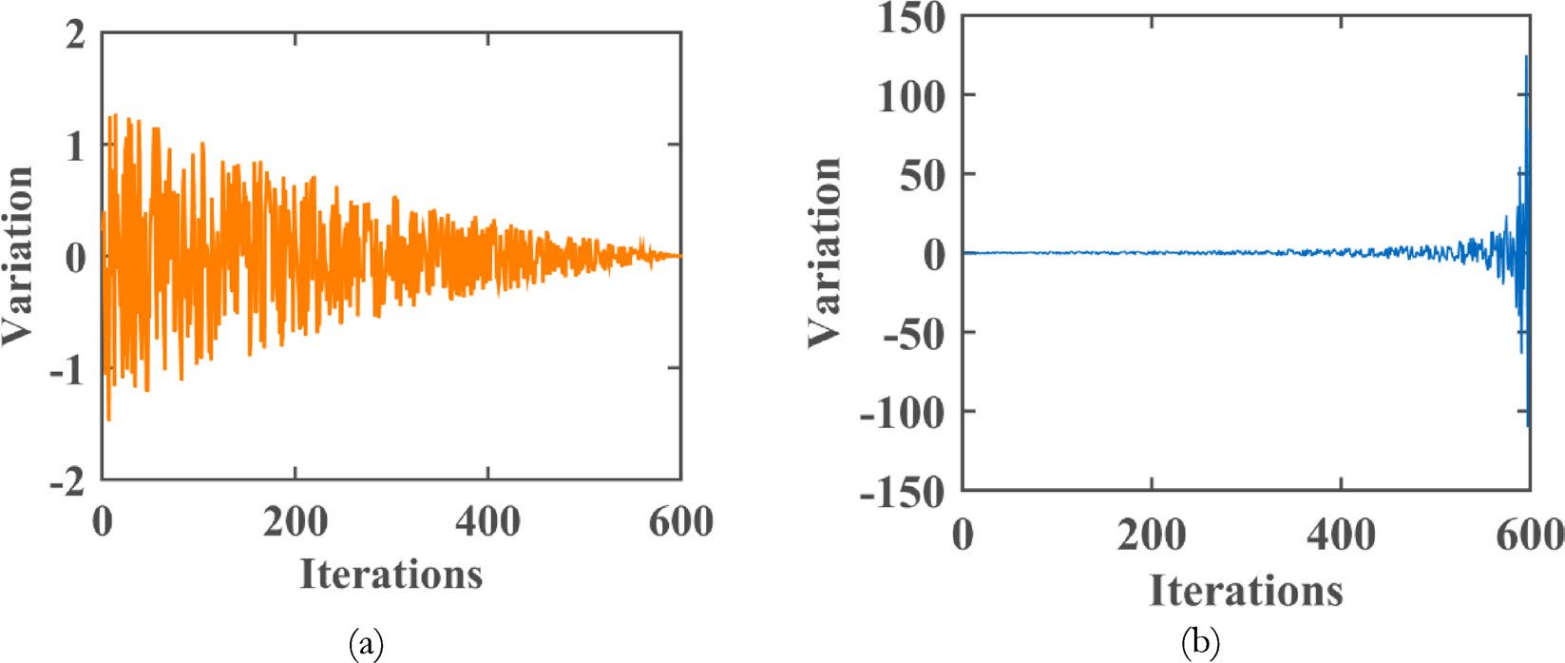
Refraction parameters in the second half refraction strategy: (**a**)Trends of $\:{Para}_{1}$ (**b**) Trends of $\:{Para}_{2}$^51^.

As illustrated in Fig. 15a, the value of $\:{Para}_{1}$​ oscillates randomly within the range $\:[-\text{2,2}]$ and converges toward zero as the number of iterations increases. Conversely, Fig. 15b shows that $\:{Para}_{2}$ exhibits random oscillations between [−150,150], with a general upward trend as iterations progress.

It is observed that both parameters initially have large values. To enhance the algorithm’s efficiency, these parameters are scaled to the standardized interval $\:\left[\text{0,1}\right]$. The oscillatory nature of $\:{Para}_{2}$​, particularly in the later stages of the FATA, improves its ability to escape local optima, thereby enhancing the robustness of the search process. Standardizing these parameters ensures smoother progression and better performance in optimization tasks.

The total internal reflection phase marks the culmination of the light propagation process in the formation of the mirage phenomenon. This stage occurs when the angle of refraction reaches a threshold where further refraction becomes impossible, causing the light to reflect entirely within the medium of inhomogeneous density.

**Fig. 16:**
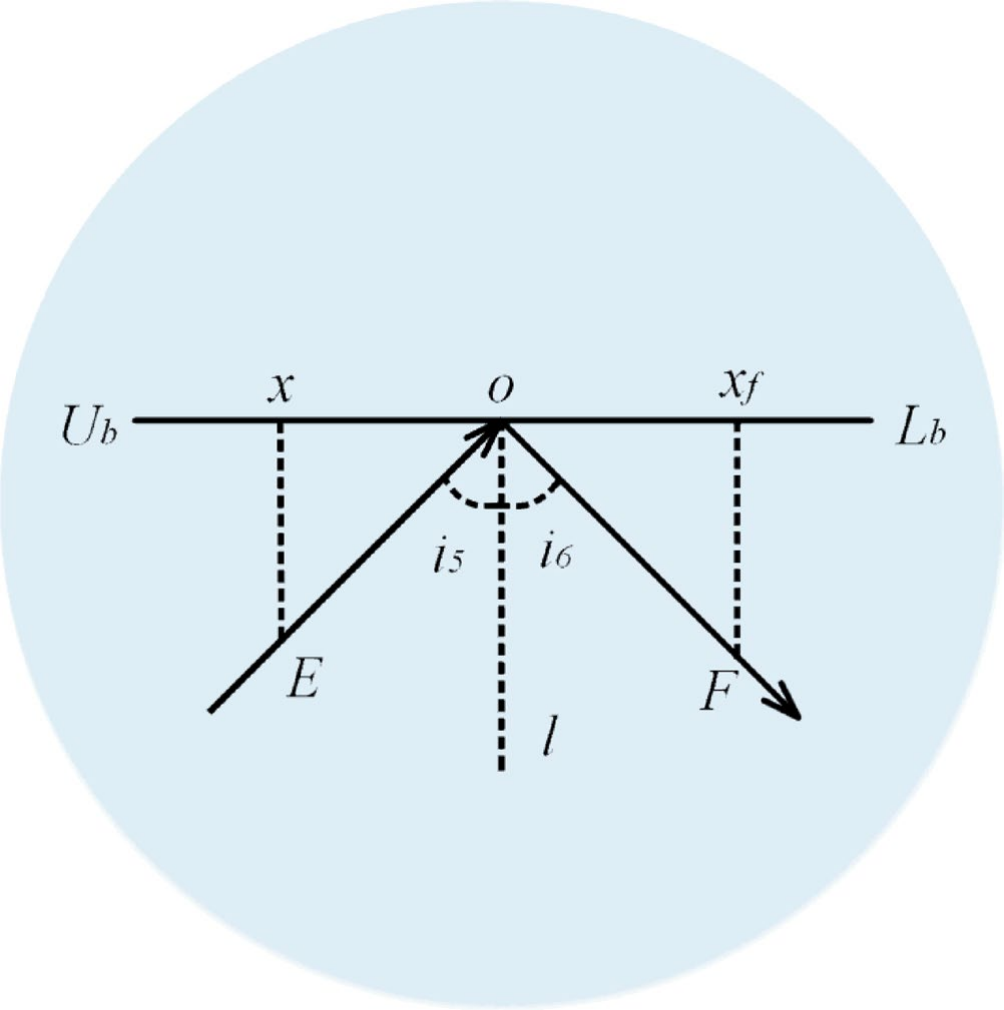
Total internal reflection process of light^51^.

This principle is implemented in the FATA as a total internal reflection strategy, which encourages the population to explore in the opposite direction, thereby enhancing diversity and exploration. As shown in Fig. 16, the angle of incidence ($\:{i}_{5}$​) equals the angle of reflection ($\:{i}_{6}$​), adhering to the laws of reflection.

In the Fig. 16:

- $\:O\left({x}_{0},0\right)$ denotes the center point of the interval ($\:[{L}_{b},{U}_{b}]$)., where $\:{L}_{b}$​ and $\:{U}_{b}$​ represent the lower and upper bounds of the search space.
- $\:E$ and $\:F$ represent the vertical distances of the incident and refracted light rays from the horizontal plane, respectively.

The total internal reflection strategy modifies the light individual ($\:x$) into a new individual ($\:{x}^{next}$) that explores the search space in the reverse direction. This mechanism increases the algorithm’s ability to identify global optima by overcoming stagnation in local optima. Equations (14–17) detail the mathematical formulation of this strategy, ensuring precise adjustments based on the geometry of the light propagation and reflection process.

42 $$\:{x}^{next}={x}_{f}=0.5\bullet\:\left({\upalpha\:}+1\right)\left({U}_{b}+{L}_{b}\right)-{\upalpha\:}x$$

43 $$\:{\upalpha\:}=\frac{F}{E}$$

44 $$\:{{x}_{0}-x}_{f}=\frac{F\bullet\:\left(x-{x}_{0}\right)}{E}$$

45 $$\:{x}_{0}=\frac{{U}_{b}-{L}_{b}}{2}+{L}_{b}=\frac{{U}_{b}+{L}_{b}}{2}$$

The individual $\:{x}_{f}$ is generated through the total internal reflection strategy. The parameter $\:{\upalpha\:}$ represents the reflectance of this strategy and governs the transformation pattern of the light individual. When $\:{\upalpha\:}$ exceeds 1, the new individual $\:{x}^{next}$ crosses the defined boundary, with $\:{\upalpha\:}$ constrained to the interval $\:\left[\text{0,1}\right]$. The specifics of $\:{\upalpha\:}$ ‘s value will be explored further in Sect. 4.2. Here, $\:{U}_{b}$ and $\:{L}_{b}$​ denote the upper and lower boundaries of the individual’s position, respectively.

**Fig. 17:**
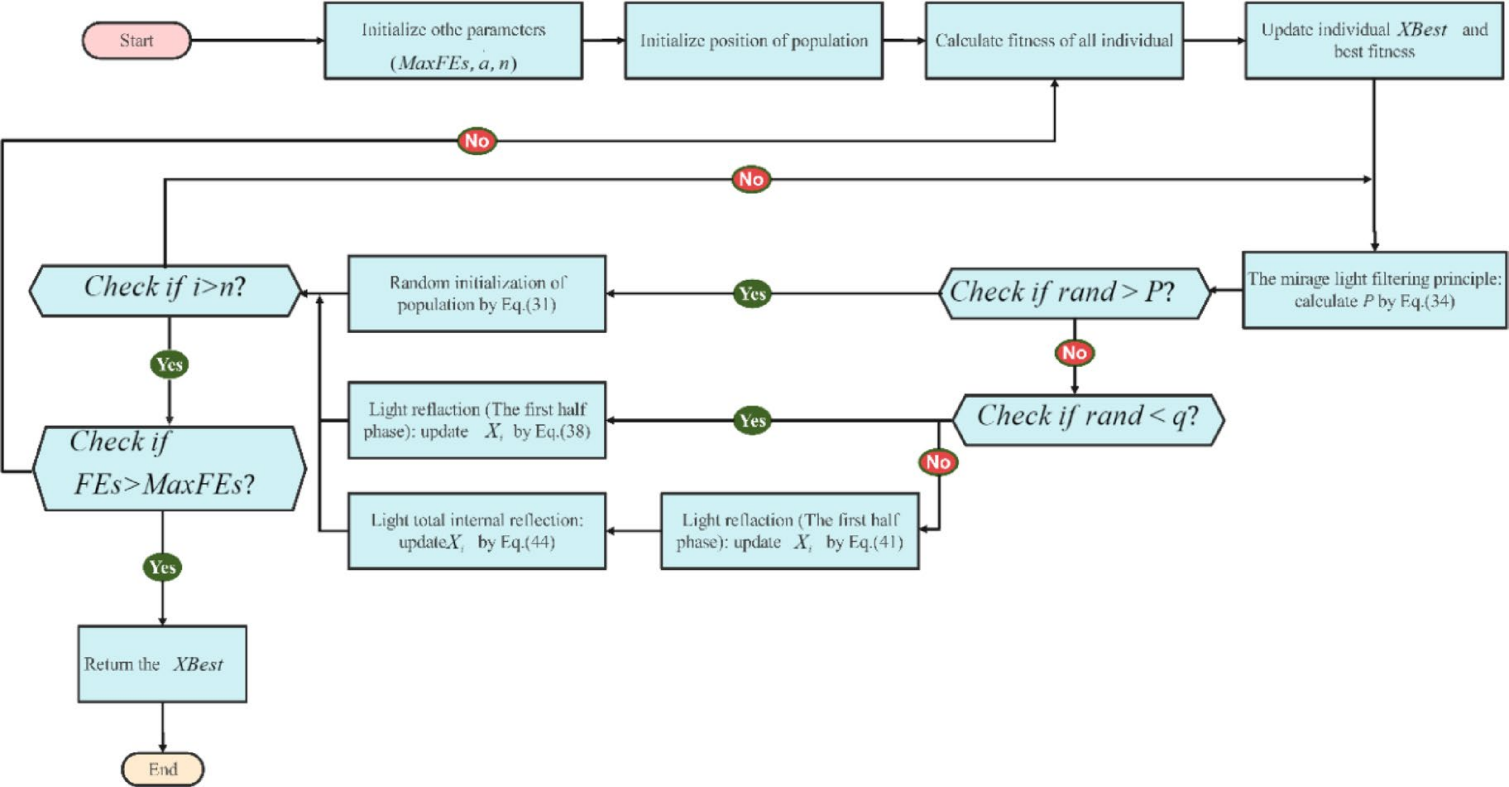
The flowchart of the FATA^51^.

To provide a comprehensive understanding of FATA, Algorithm 2 presents its pseudocode, while Fig. 17 illustrates the workflow, highlighting the two core population updating mechanisms. The algorithm follows a structured process consisting of population initialization, parameter configuration, and an iterative evolution phase. Within this phase, the computational burden of both the mirage light filtering and light refraction strategies is primarily determined by the number of iterations, resulting in a time complexity of $\:{\rm\:O}\left(n\right(\:MaxFEs\:\bullet\:\:d\left)\right)$.

**Algorithm 2:**
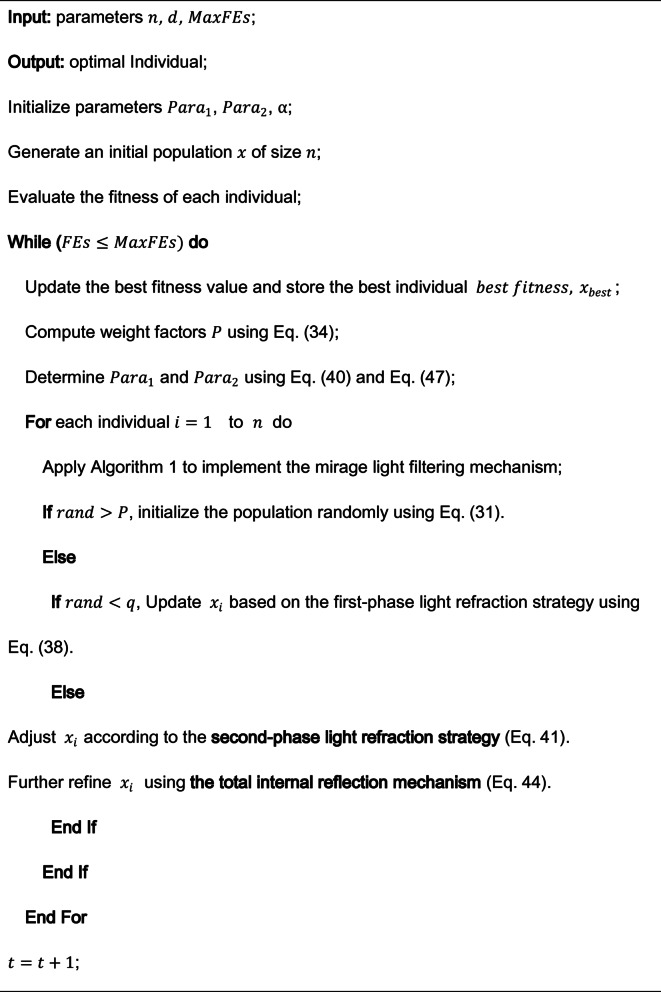
Pseudocode of FATA

## Simulation and experimental results

In this study, the control of a two-area power system powered by three different energy sources was investigated using PI, PIDn, FOPI, and PPIDn controllers. The parameters of these controllers were optimized using GJO, ECO, ESC, and FATA algorithms.

**Fig. 18:**
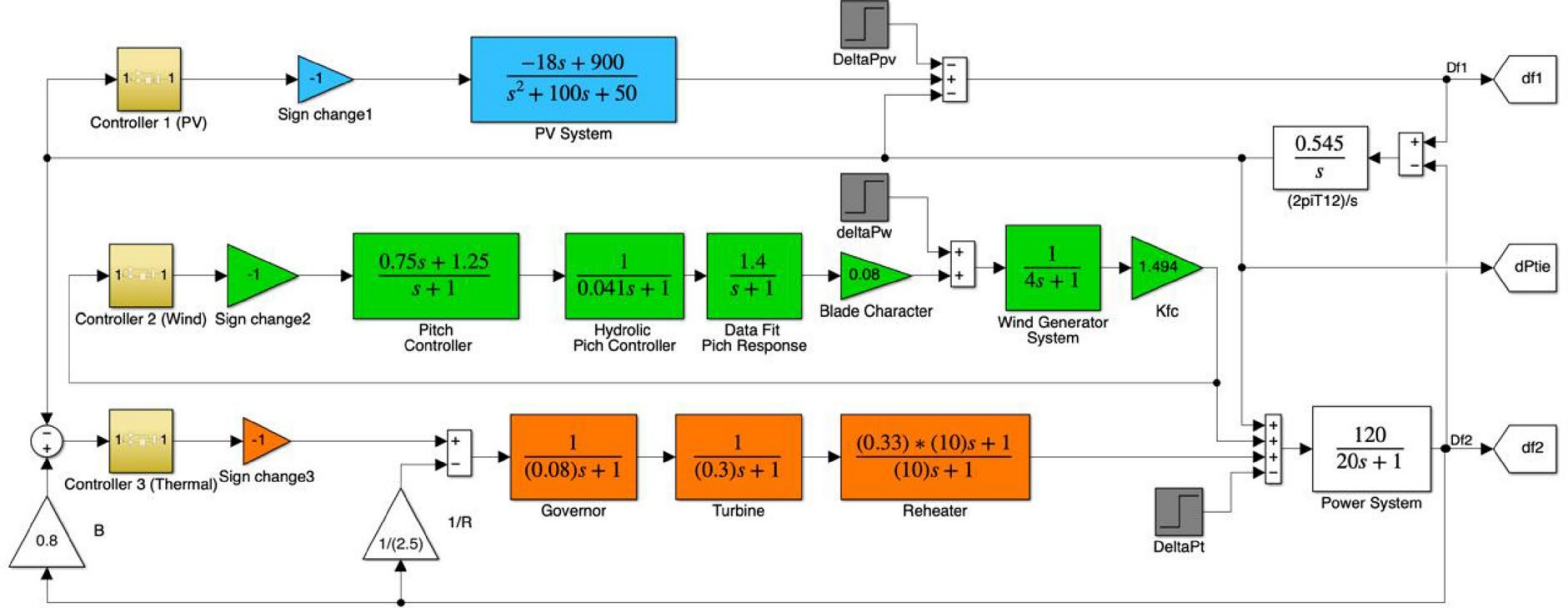
Simulation system.

Subsequently, the obtained results were validated through OPAL-RT, and a comparative analysis was conducted between the simulation outcomes and experimental results. The simulated system is illustrated in Fig. 18.

The mapping of the controllers is defined as Controller 1 – PV system, Controller 2 – Wind system, and Controller 3 – Thermal system. This configuration ensures clear identification and consistency with the corresponding parameter tables (Tables 7, 8, 9, 10, 11, 12, 13, 14, 15 and 16), where the same controller indices are used for comparative and optimization analyses.

**Table 7:** PI controller parameters with ITAE values.

Controller	Opt. Alg.	K_p1_	K_i1_	K_p2_	K_i2_	K_p3_	K_i3_	ITAE
**PI**	**GJO**	0.5251	0.1310	3.9961	−0.0146	2.5244	0.1438	**1.5849**
	ECO	0.49747	0.3525	3.3686	−0.021695	2.6645	0.71119	2.7082
	ESC	0.66383	0.14652	3.9752	−0.0026187	2.4988	0.22543	1.7212
	FATA	0.95732	0.082446	1.7621	−0.0072356	1.1953	1.1022	4.959

**Table 8:** PIDn controller 1(PV) parameters.

Controller 1	Optimization Algorithms	K_p1_	K_i1_	K_d1_	*n*_1_
PIDn	GJO	2.9666	0.2723	2.4420	145.8440
	ECO	0.9838682	0.3387465	0.4080806	194.7095
	ESC	3.509963	0.2967109	1.076755	95.23372
	FATA	3.72744	1.787188	1.018562	155.9837

**Table 9:** PIDn controller 2 (Wind) parameters.

Controller 2	Optimization Algorithms	K_p2_	K_i2_	K_d2_	*n*_2_
PIDn	GJO	0.1865	5.8383e-04	−0.0010	1.5955
	ECO	−0.1158584	0.1360314	−0.8529435	2.419207
	ESC	0.471755	−0.04236771	3.153995	105.7223
	FATA	2.114036	−0.007076795	0.5647366	144.5859

**Table 10:** PIDn controller 3 (Thermal) parameters with ITAE values.

Controller 3	Optimization Algorithms	K_p3_	K_i3_	K_d3_	*n*_3_	ITAE
**PIDn**	GJO	4	4	0.4301	9.0091	0.1958
	ECO	3.999821	3.999953	0.3454577	179.8215	0.2531
	ESC	3.977549	3.928608	0.4525752	11.23297	0.19669
	**FATA**	4	3.973674	0.524713	7.918286	**0.18676**

**Table 11:** FOPI controller 1 and 2 parameters.

Controller	Optimization Algorithms	K_p1_	K_i1_	λ_1_	K_p2_	K_i2_	λ_2_
FOPI	GJO	−0.0027	0.8845	0.4591	3.0071	−0.2188	0.3792
	ECO	−0.39365	1.5131	0.29532	−1.2835	2.8498	0.0001
	ESC	0.77817	0.51205	0.33906	2.3735	−0.2992	0.22255
	FATA	0.33822	0.23075	0.40761	0.89628	0.0073297	0.54136

**Table 12:** FOPI controller 3 parameters and ITAE values.

Controller	Optimization Algorithms	K_p3_	K_i3_	λ_3_	ITAE
**FOPI**	GJO	2.3013	0.2927	2.7284e-04	2.1444
	**ECO**	3.8094	−0.83418	0.49918	**2.0176**
	ESC	2.4733	−0.35033	0.52696	2.7095
	FATA	0.38526	0.892	0.49146	7.4598

**Table 13:** PPIDn controller 1(PV) parameters.

Controller 1	Optimization Algorithms	K_p1_	K_i1_	K_d1_	*n*_1_	K_prd1_	Kh_1_
PPIDn	GJO	0.9840	0.2482	3.0399	150.5947	0.0150	0.0299
	ECO	4	1.599685	3.750207	108.2514	3.877172	0
	ESC	3.924731	1.062002	3.218801	115.3733	0.0484639	0.1878636
	FATA	1.170467	0.2721576	1.089585	53.07538	0	0

**Table 14:** PPIDn controller 2 (Wind) parameters.

Controller 2	Optimization Algorithms	K_p2_	K_i2_	K_d2_	*n*_2_	K_prd2_	Kh_2_
PPIDn	GJO	−0.0331	−0.0477	5.5685e-05	1.4783	2.0418	1.1120
	ECO	4	0.006105196	−3.256132	99.70377	3.887082	0
	ESC	3.992627	−0.01261917	3.945147	1.001308	0.01774135	0.0471736
	FATA	1.184828	0.2753649	0.9450793	116.2314	4	1.745256

**Table 15:** PPIDn controller 3 (Thermal) parameters.

Controller 3	Optimization algorithms	K_p3_	K_i3_	K_d3_	*n*_3_	K_prd3_	Kh_3_
**PPIDn**	GJO	4	4	0.7174	10.9674	0	0.0063
	ECO	3.608775	3.309643	0.5120614	152.9706	0.006157692	0.2757894
	ESC	3.979733	3.990961	0.8278113	11.4859	0.07643719	1755.635e-6
	FATA	1.633781	4	1.895669	193.2236	1.867529	0

**Table 16:** PPIDn controller ITAE parameters.

Controller	Optimization algorithms	ITAE
**PPIDn**	GJO	0.6218
	ECO	0.60473
	**ESC**	**0.3896**
	FATA	1.6889

### Parameters

In this study, four different controllers were optimized using four distinct algorithms, resulting in a total of 16 optimization scenarios. The optimization process was conducted with a population size of 50 and 100 iterations for each algorithm.The solar panel model was based on an ambient temperature of 27 °C and a solar irradiance of 1000 W/m². The wind energy system consisted of 55 wind turbines. The simulations were performed in the MATLAB/Simulink environment using transfer function-based modeling. Some of the wind turbine parameters listed in Table 17 are provided for informational purposes.

**Table 17:** System parameter values used in simulation^43^.

Variable	Value	Unit	Unite
*a*	−18	---	PV Solar Power
*b*	900	---	PV Solar Power
*c*	100	---	PV Solar Power
*d*	50	---	PV Solar Power
*D*	43	meter	Wind Turbine
*a*_*s*_	1452	m^2^	Wind Turbine
*ρ*	1.225	kg/m^3^	Wind Turbine
*H*	40	m	Wind Turbine
*ω*_*r*_	27.2/18.1	rpm	Wind Turbine
*P*_*wt*_	600	kW	Wind Turbine
*C*_*P*_	0.59	---	Wind Turbine
*K*_*p1*_	1.25	p.u. MW	Wind Energy
*K*_*p2*_	1	p.u. MW	Wind Energy
*K*_*p3*_	1.4	p.u. MW	Wind Energy
*K*_*pc*_	0.08	---	Wind Energy
*K*_*fc*_	1.494	---	Wind Energy
*T*_*p1*_	0.6	s	Wind Energy
*T*_*p2*_	0.041	s	Wind Energy
*T*_*p3*_	1	s	Wind Energy
*T*_*w*_	4	s	Wind Energy
*P*_*R*_	200	MW	Thermal System
*P*_*L*_	100	MW	Thermal System
*R*_*1*_	2.5	Hz/p.u. MW	Thermal System
*B*	0.8	p.u. MW/Hz	Thermal System
*K*_*gov*_	1	p.u. MW	Thermal System
*T*_*gov*_	0.08	s	Thermal System
*K*_*tur*_	1	p.u. MW	Thermal System
*T*_*tur*_	0.3	s	Thermal System
*K*_*reh*_	0.33	p.u. MW	Thermal System
*K*_*ps*_	120	Hz/p.u. ΜΩ	Thermal System
*T*_*ps*_	20	s	Thermal System
*T*_*reh*_	10	s	Thermal System
*2φT*_*12*_	0.545	p.u.	Thermal System
*a*_*12*_	−1	---	Thermal System
f	60	Hz	Thermal System

The values of the parameters used in the simulations are summarized in Table 17.

Here, *R* is the governor speed regulation constant and *B* shows the frequency bias parameter, *H* is hub height and *K*_*pc*_ is blade character^43^.

The lower and upper limits of the controllers used in the study are presented in Table 18. The optimization algorithms search for optimal parameters within these predefined ranges.

**Table 18:** The controller parameters limits.

Variable	Lower	Upper
*K*_*P*_	−4	4
*K*_*I*_	−4	4
*K*_*D*_	−4	4
*K*_*prd*_	0	4
*K*_*h*_	0	2
*n*	1	200
*λ*	0.0001	1

To establish these limits, a preliminary analysis was conducted. Initially, the best possible values were determined using the Ziegler-Nichols method and trial-and-error approaches. The search range was then set to encompass these values. Additionally, parameter ranges from previous studies in the literature were considered when defining the search space.

The simulation was conducted by applying a 10% load increase to each region in the system.

### Simulation

In the simulation studies, PI, PIDn, FOPI, and PPIDn controllers were implemented. The parameters of each controller were optimized using GJO, ECO, ESC, and FATA algorithms within the search space defined in Table 18, aiming to determine the best possible values. A total of 16 different simulations were conducted.

For each optimization algorithm, the population size was set to 50, and the number of iterations was fixed at 100. The performance of the optimization algorithms was evaluated using the ITAE criterion as a reference.

The best parameter values obtained for PI controllers using different optimization algorithms are presented in Table 7. The lowest ITAE value, 1.5849, was achieved using the GJO algorithm.

The optimization results for the PIDn controller are presented collectively in Tables 8, 9 and 10. The data is divided into three sections:

- Table 8 displays the results for the solar energy system controller.
- Table 9 presents the results for the thermal energy system controller.
- Table 10 provides the results for the wind energy system controller.

Among these, the best ITAE value was achieved using the FATA algorithm, with a value of 0.18676.

The optimization results for the FOPI controller are presented in Tables 11 and 12.

- Table 11 provides the optimized parameters for FOPI controllers 1 and 2.
- Table 12 lists the parameters for FOPI controller 3 along with the corresponding ITAE values.

Among the tested algorithms, the lowest ITAE value (2.0176) was obtained using the ECO algorithm.

The optimization results for the PPIDn controller are presented in Tables 13, 14, 15 and 16.

- Table 13 provides the optimized parameters for the solar energy system controller.
- Table 14 presents the parameters for the thermal energy system controller.
- Table 15 lists the parameters for the wind energy system controller.
- Table 16 summarizes the ITAE values obtained using different optimization algorithms for the PPIDn controller.

Among the tested algorithms, the ESC algorithm achieved the lowest ITAE value of 0.3896.

**Fig. 19:**
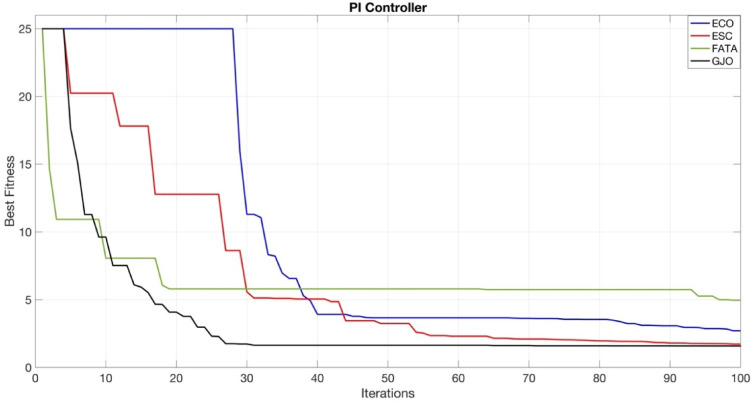
Convergence curve for PI controller.

**Fig. 20:**
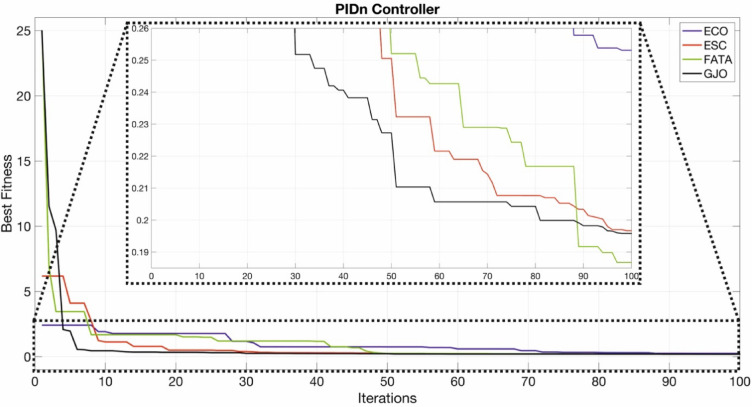
Convergence curve for PIDn controller.

**Fig. 21:**
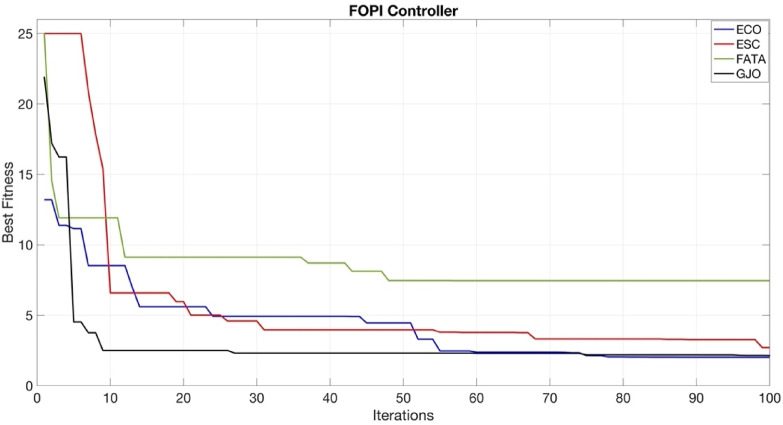
Convergence curve for FOPI controller.

**Fig. 22:**
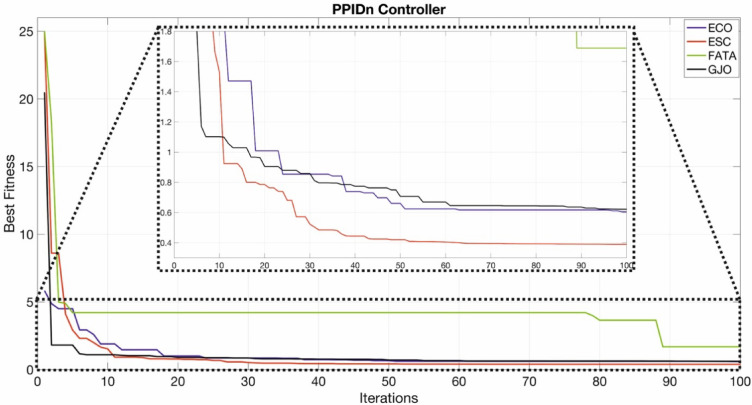
Convergence curve for PPIDn controller.

The convergence curves of the PI, PIDn, FOPI, and PPIDn controllers are illustrated in Figs. 19, 20, 21 and 22.

- Figures 20 and 22 provide a more detailed visualization of the curves for better clarity.
- Figs. 19 and 21 do not require detailed plotting, as the general trends are sufficiently visible.

In Fig. 19, the GJO algorithm achieved the lowest ITAE value, which, as shown in Table 7, is 1.5849.

Figure 20 presents the optimization results for the PIDn controller. Among the tested algorithms, the FATA algorithm achieved the lowest ITAE value of 0.18676, indicating superior performance. This trend is clearly observed in the magnified section of the figure and is further validated by the results in Table 10.

Figure 21 illustrates the optimization results for the FOPI controller using ECO, ESC, FATA, and GJO algorithms. The vertical axis represents the best fitness value, while the horizontal axis shows the number of iterations. Among the tested algorithms, ECO achieved the lowest ITAE value of 2.0176, indicating the best optimization performance for this controller. As seen in the figure, GJO (black) and ECO (blue) exhibit rapid initial convergence, whereas FATA (green) stagnates at a higher fitness level, suggesting ineffective parameter tuning. The ESC (red) algorithm shows a moderate convergence rate, stabilizing at a slightly higher ITAE value than ECO. The results indicate that ECO effectively balances exploration and exploitation, leading to faster convergence and lower ITAE values. In contrast, FATA struggles to refine the solution after early iterations, possibly due to its search strategy. This finding aligns with previous studies where ECO has demonstrated robust performance in fine-tuning controller parameters.

Figure 22 illustrates the optimization results for the PPIDn controller using ECO, ESC, FATA, and GJO algorithms. The best fitness values are plotted against the number of iterations, with a zoomed-in section highlighting the finer details of the convergence behavior. Among the tested algorithms, ESC achieved the lowest ITAE value of 0.3896, indicating superior optimization performance. As seen in the figure, ESC (red) exhibits rapid convergence and stabilizes at a lower fitness value compared to the other algorithms. ECO (blue) and GJO (black) also show stable convergence, but at slightly higher ITAE values. FATA (green), on the other hand, stagnates at a higher fitness level, suggesting suboptimal tuning. The results suggest that ESC effectively balances exploration and exploitation, allowing it to converge to an optimal solution more efficiently. The detailed section in Fig. 22 further highlights this advantage by showcasing the faster fitness improvement of ESC compared to the other methods. These findings reinforce the effectiveness of ESC in optimizing PPIDn controller parameters for this system.

Table 19 presents the optimized PIDn controller parameters obtained using the FATA algorithm, as demonstrated in Tables 8 and 9, and 10. These parameters were derived from 16 different simulation studies, where the FATA algorithm achieved the lowest ITAE value of 0.18676.

As seen in Table 19, the optimal values for K_p_​, K_i_, K_d_, and n were determined for each region. These values were fine-tuned to enhance system performance, ensuring improved frequency stability and dynamic response. The low ITAE value indicates the effectiveness of FATA in optimizing PIDn controllers, making it a strong candidate for load frequency control in multi-area power systems.

**Fig. 23:**
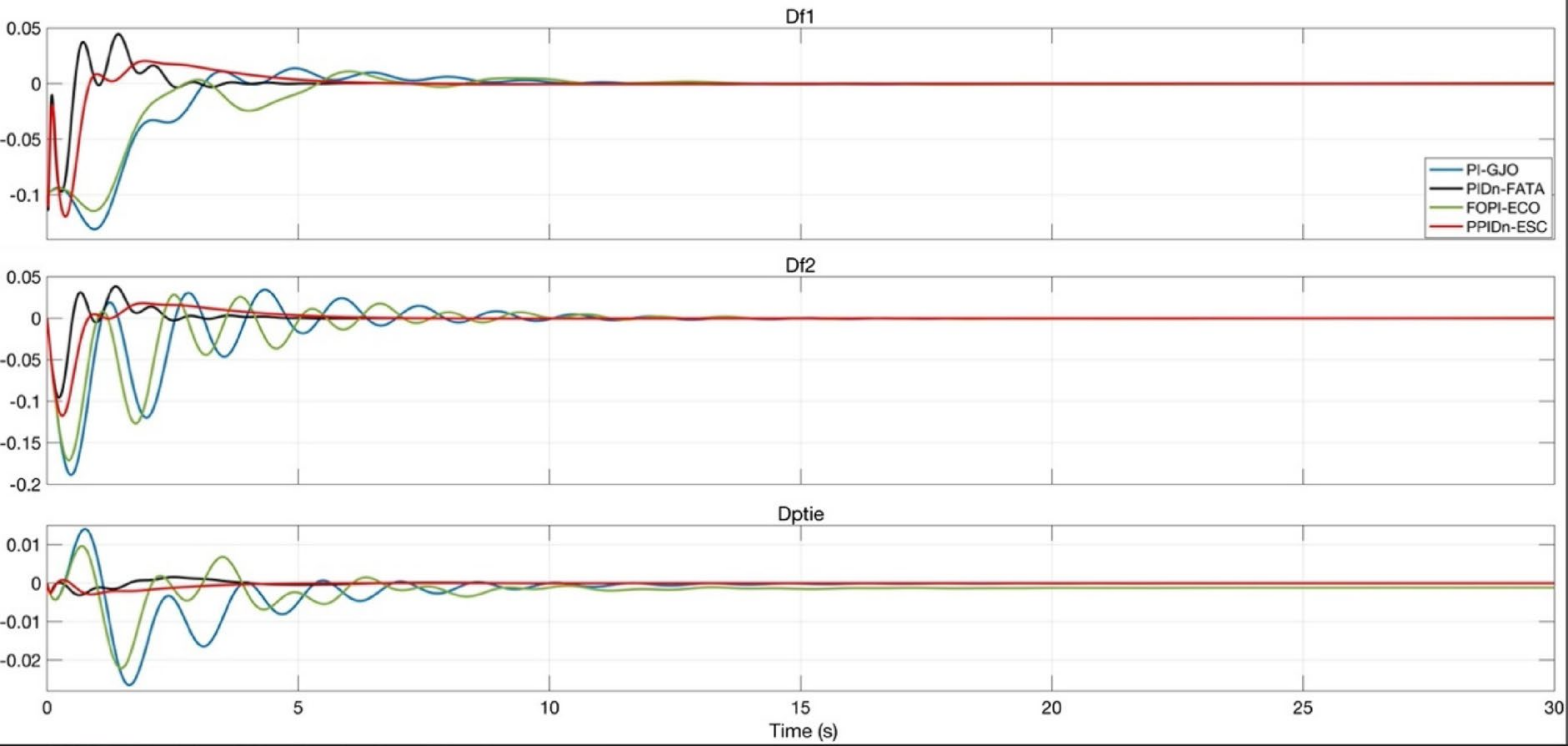
System responses with the best-tuned parameters for PI, PIDn, FOPI, and PPIDn controllers.

Figure 23 presents the system responses obtained using the best-optimized parameters for each controller (PI, PIDn, FOPI, and PPIDn). The figure illustrates the frequency deviations in each region (Δf_1_​ and Δf_2_​) as well as the tie-line power deviation (ΔP_tie_) over the time. As shown, the PPIDn-ESC controller (red) provides the fastest settling time and minimal oscillations, indicating superior performance in stabilizing frequency fluctuations. The PIDn-FATA (green) and FOPI-ECO (blue) controllers exhibit relatively higher oscillations but still converge to a stable state. In contrast, the PI-GJO controller (black) shows the largest initial deviations and prolonged oscillations, suggesting slower stabilization. These results confirm that ESC-optimized PPIDn achieves the best dynamic response, effectively minimizing frequency deviations and ensuring system stability in a multi-area power system.

**Fig. 24:**
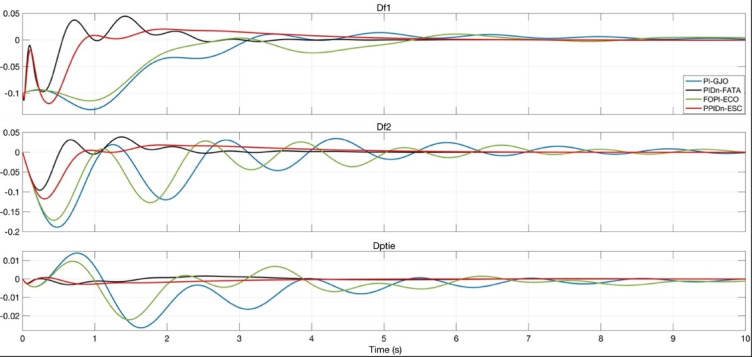
Detailed system responses with the best-tuned parameters for PI, PIDn, FOPI, and PPIDn controllers.

Figure 24 provides a more detailed view of the system responses, illustrating the frequency deviations Δf_1_​ and Δf_2_​ in each region and the tie-line power deviation ΔP_tie_ over a shorter time interval.

Compared to Fig. 23, this zoomed-in representation allows for a clearer analysis of the initial transient behavior and oscillatory characteristics of each controller. The differences in settling time, peak overshoot, and damping performance among the PI-GJO, PIDn-FATA, FOPI-ECO, and PPIDn-ESC controllers can be observed more precisely.

Table 19 presents a comparative analysis of the settling time (T_S_), undershoot (U_S_), and overshoot (O_S_) values for the frequency deviations shown in Fig. 24, based on the optimized parameters of each controller.

**Table 19:** Comparison of the best results obtained with optimization algorithms for all controllers in terms of T_S_, U_S_, and O_S_.

Cont.	Alg.	T_S_ (Settling Time)			U_S_ (Undershoot)			O_S_ (Overshoot)		
		Δf_1_	Δf_2_	Δf_tie_	Δf_1_	Δf_2_	Δf_tie_	Δf_1_	Δf_2_	Δf_tie_
**PI**	GJO	12	13	12	−0.13	−0.18	−0.0285	0.0139	0.0345	0.014
**PIDn**	FATA	**4**	**4**	**4**	**−0.09**	**−0.09**	**−0.0023**	0.05	0.0384	0.00153
**FOPI**	ECO	11	12	---	−0.11	−0.165	−0.0215	**0.011**	0.0283	0.01
**PPIDn**	ESC	6	6	**4**	−0.12	−0.12	−0.0025	0.02	**0.018**	**0.00077**

Among the tested controllers, the PIDn controller optimized with FATA achieved the best results in terms of settling time and undershoot, demonstrating faster stabilization and minimal deviations. On the other hand, the PPIDn controller optimized with ESC exhibited the lowest overshoot values, indicating superior damping performance. The FOPI controller optimized with ECO provided the best overshoot performance only for the first region’s frequency deviation. Meanwhile, the PI controller failed to achieve competitive results across any of the evaluated criteria, performing worse than the other controllers.

### Experimental results

The OPAL-RT OP5707 platform was utilized to validate the real-time performance of the proposed controllers. This hardware-based environment allows the assessment of control algorithms under realistic conditions, including finite sampling intervals, communication delays, and numerical precision effects that are not fully captured in offline simulations. Such validation ensures that the proposed FATA-optimized controller is not only theoretically efficient but also practically reliable for real-world power system applications.

**Fig. 25:**
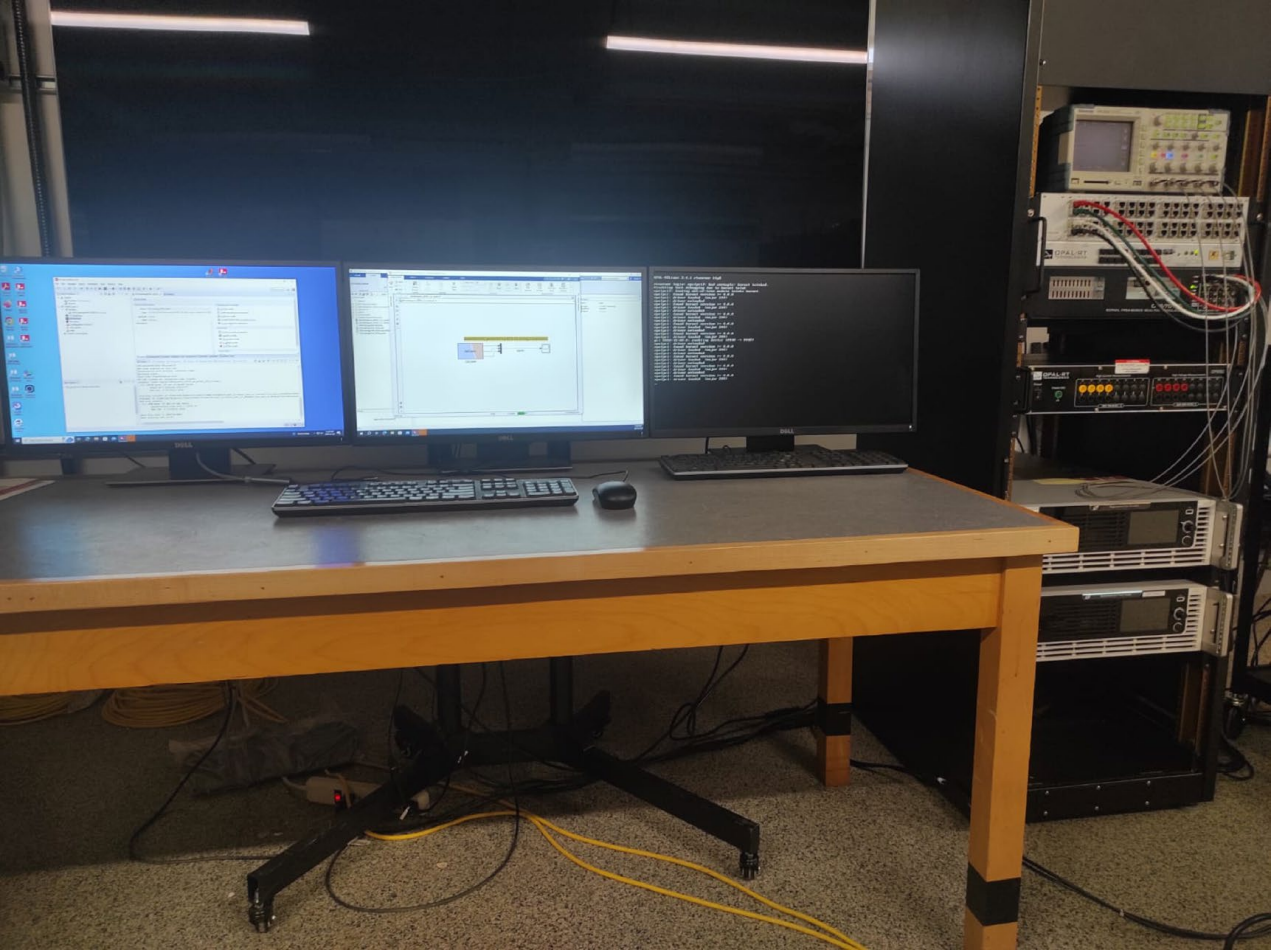
OPAL-RT experimental setup.

Among all the evaluated controllers, the PIDn controller achieved the best performance. Its parameters were optimized using the FATA algorithm, as presented in Table 7. To verify the reliability of these results, the optimized parameters were implemented on the OPAL-RT platform, and the experimental outcomes were compared with the simulation results. The experimental setup used for real-time validation is shown in Fig. 25.

**Fig. 26:**
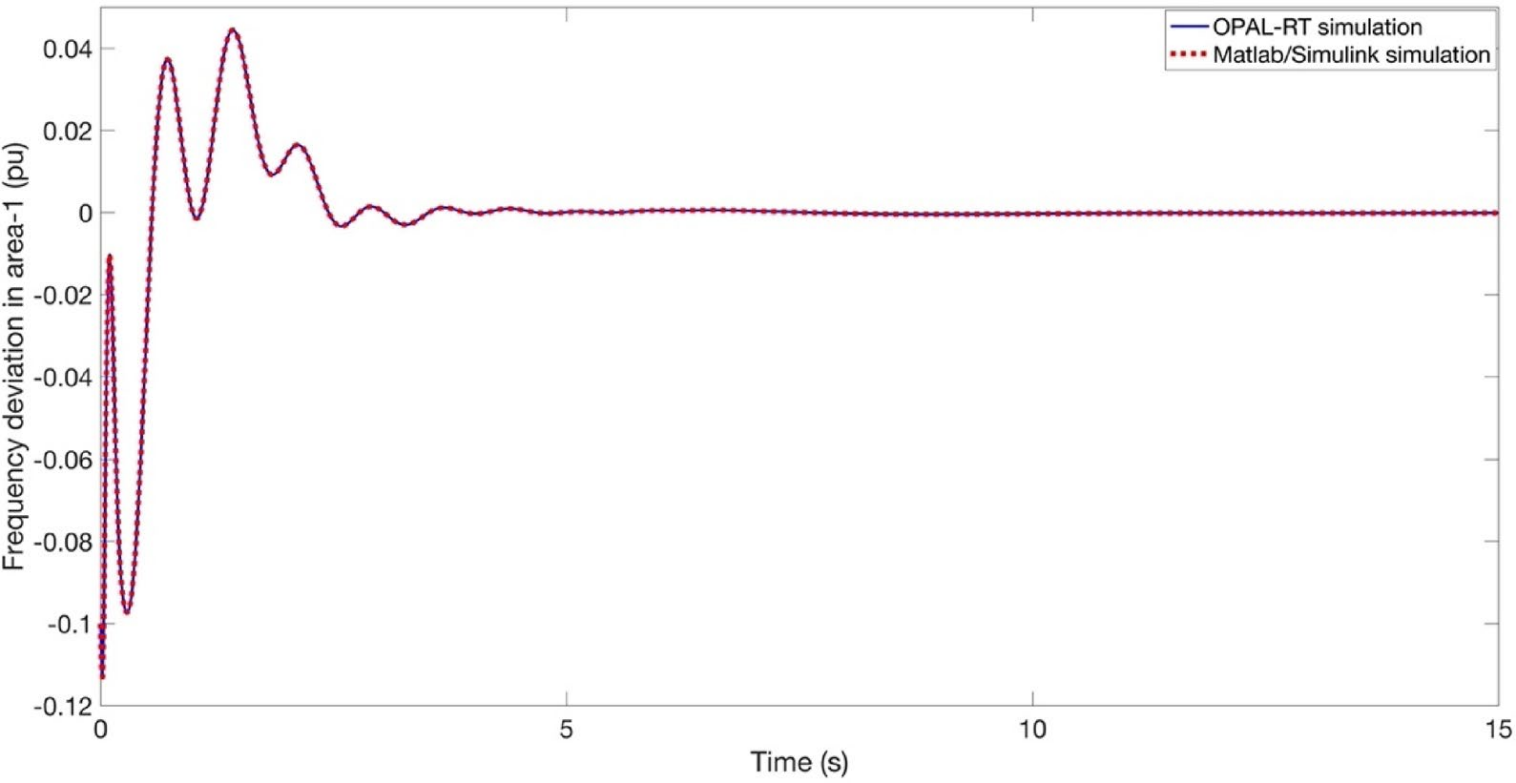
OPAL-RT result of Δf_1_.

**Fig. 27:**
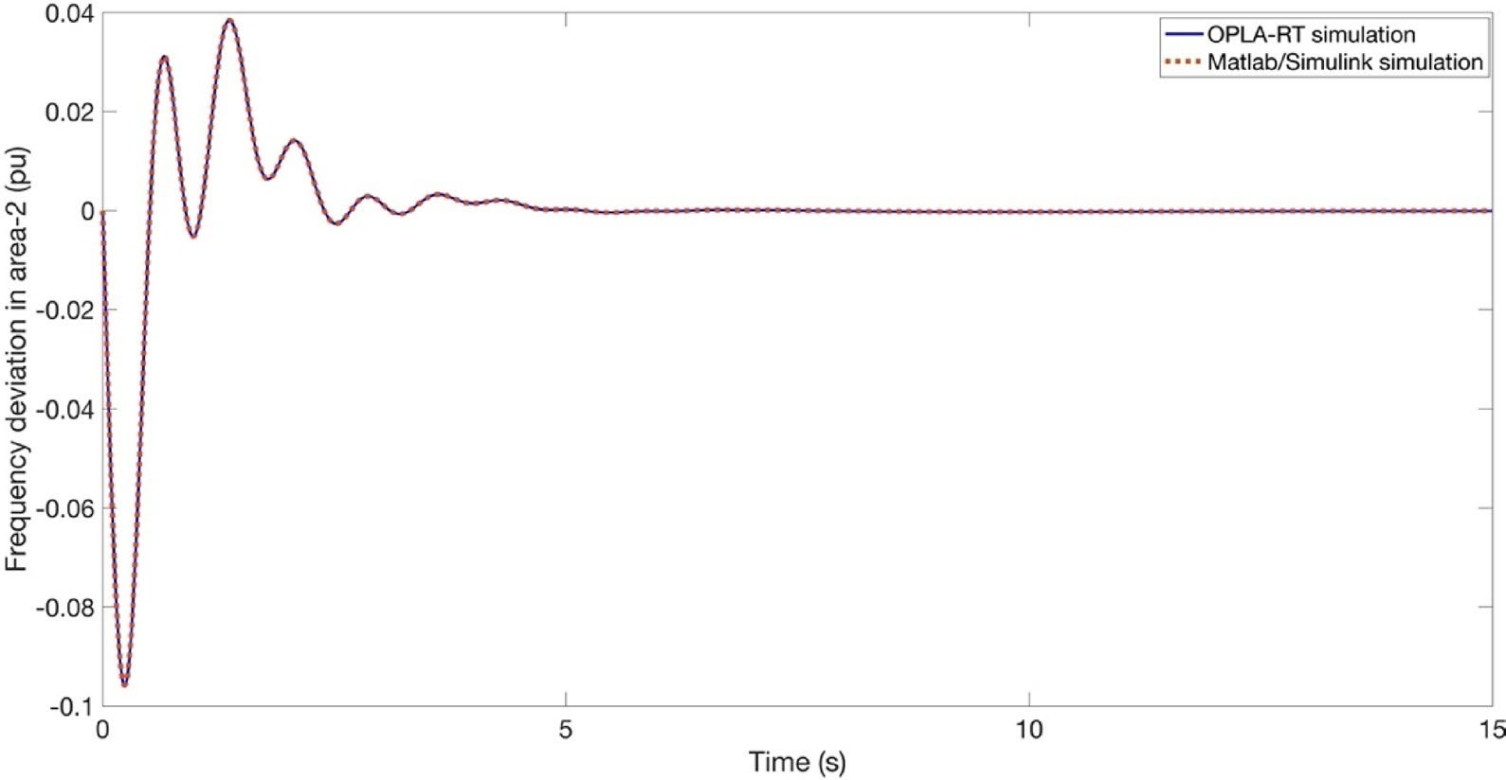
OPAL-RT result of Δf_2_.

**Fig. 28:**
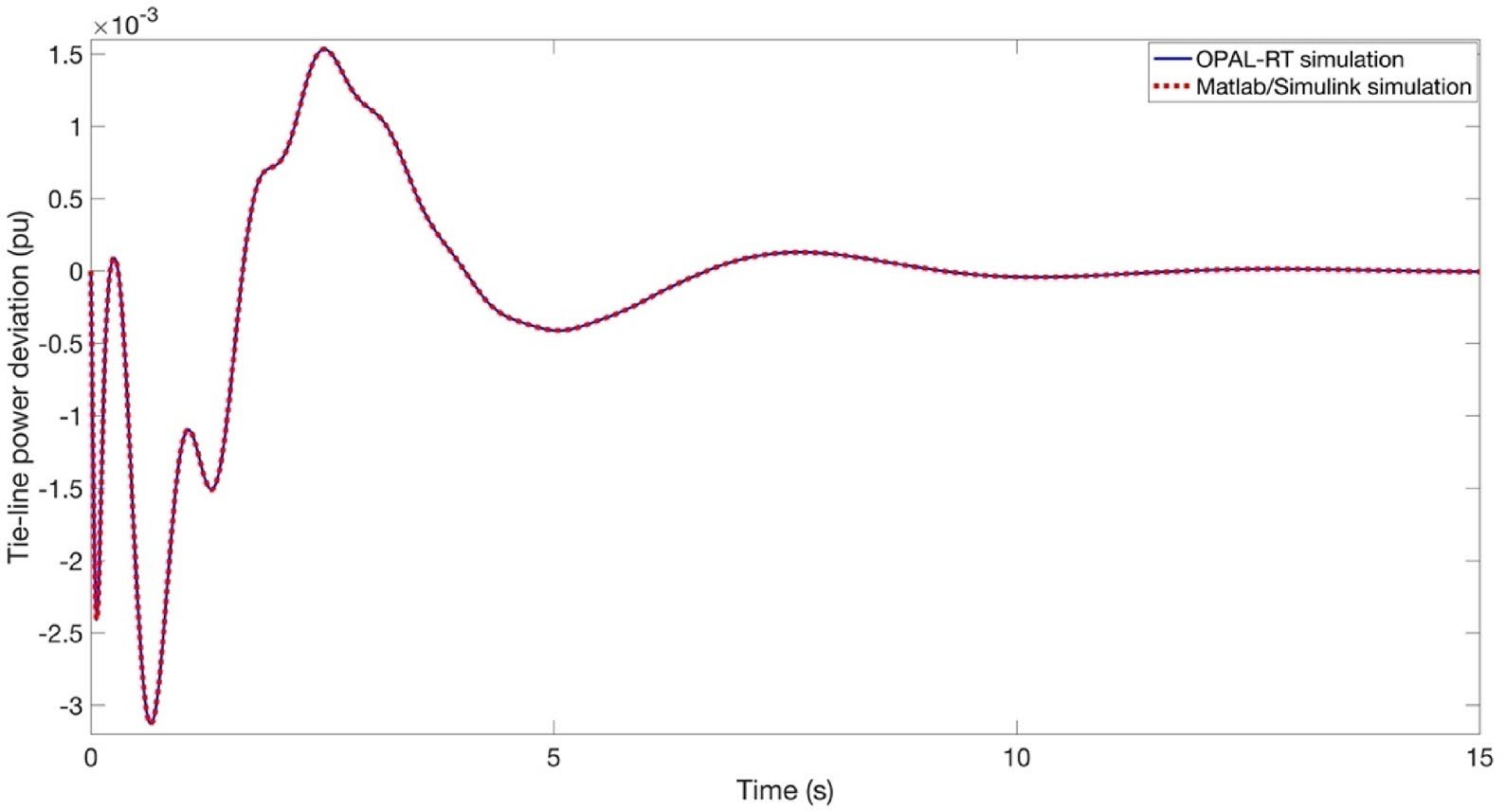
OPAL-RT result of ΔP_tie_.

The objective of this section is to validate the simulation outcomes Matlab/Simulink program in the context of real-time implementation of the proposed approach. For this purpose, this process is illustrated as shown in Fig. 25. It is imperative to acknowledge that the outcomes of the OPAL-RT investigations are influenced by intrinsic delay and error components that are not present in conventional offline implementations. As shown in Figs. 26 and 27, and 28, the time domain responses of the Δf_1_​, Δf_2_​, and ΔP_tie_ based on the FATA-PIDn controller are depicted, with these responses being based on the real-time simulator OPAL-RT. As evidenced by Fig. 25, the results obtained from the MATLAB simulation software demonstrate a notable degree of similarity to those produced by the OPAL-RT real-time simulator.

Figures 26 and 27, and 28 present the experimental and simulation results obtained using the PIDn controller optimized with the FATA algorithm. These figures compare the real-time performance of the controller, demonstrating its effectiveness in practical implementation.

In Fig. 26, the Δf_1_​ in area-1 is depicted. The results from the OPAL-RT experimental setup and Matlab/Simulink simulation are shown together, indicating a strong correlation between the experimental and simulation data. This consistency validates the accuracy and reliability of the proposed control approach.

Figure 27 illustrates the Δf_2_ ​in Area 2. The experimental results obtained from the OPAL-RT platform and the Matlab/Simulink simulation show an almost identical response, as the curves overlap perfectly. This strong agreement confirms the accuracy and reliability of the proposed PIDn controller optimized with the FATA algorithm in real-time implementation.

Figure 28 illustrates the power deviation at the tie-line between the two areas. The experimental results from the OPAL-RT platform and the Matlab/Simulink simulation are perfectly aligned, demonstrating an exact match. This strong correlation confirms the accuracy and reliability of the proposed PIDn controller optimized with the FATA algorithm in maintaining frequency stability across interconnected regions.

It is again noteworthy that the OPAL-RT hardware inherently involves minor latency and dead-time effects caused by real-time signal processing and data communication. Despite these practical nonlinearities, the experimental results obtained from the OPAL-RT platform closely matched the MATLAB/Simulink simulations. Therefore, the proposed model and controller design are considered sufficiently accurate and robust for realistic operating conditions.

## Discussion

In this study, PI, PIDn, FOPI, and PPIDn controllers were implemented to regulate frequency in a two-area power system with three different energy sources. The parameters of these controllers were optimized using GJO, ECO, ESC, and FATA algorithms. Among the 16 different optimization scenarios, the PIDn controller optimized with the FATA algorithm demonstrated the best performance in simulation studies.

To validate these results, the optimized parameters were tested on the OPAL-RT real-time simulation platform. The experimental results closely matched the simulation outcomes, with overlapping response curves confirming the accuracy and reliability of the proposed control strategy.

Based on the findings, the following key conclusions can be drawn:

- Despite its more complex structure, the PPIDn controller did not outperform the PIDn controller, which achieved the best results among the tested controllers.
- The selected optimization algorithms were chosen based on their potential to effectively address frequency deviation issues in a two-area power system.
- Simulation runtime influences optimization performance, and a population size of 50 with 100 iterations was considered sufficient for the optimization process.
- Since ITAE values change over time, longer simulation durations result in higher ITAE values. Therefore, the simulation time must be carefully selected to ensure a meaningful comparison.
- The experimental validation of the results confirms the accuracy and correctness of the optimization process, demonstrating the practical applicability of the proposed method.

When the obtained results are compared with similar studies in the literature, it is observed that in the study where PI/PID/PDn-PI cascade controllers were tested, the controller parameters were optimized using the Coyote optimization algorithm. During a 30-second simulation period, the ITAE value was measured, and the best result of 1.935 was achieved with the PDn-PI controller. In the present study, however, a significantly improved ITAE value of 0.18676 was obtained^48^.

In conclusion, for the considered power system, the PIDn controller optimized with FATA exhibited the best overall performance, effectively improving frequency stability and system response.

## Conclusion and recommendations

Based on this study, the following recommendations are provided for researchers conducting similar work:

- The most effective controller cannot always be predicted in advance. In some cases, even the simplest controllers may yield the best results.
- Exploring different optimization algorithms may lead to improved outcomes. Testing alternative methods could further enhance performance.
- Increasing the population size and the number of iterations can improve optimization results. However, this also increases computational time. Therefore, unnecessary simulation time should be avoided, particularly by considering the settling time as a critical factor.
- Utilizing more advanced computing systems can significantly reduce processing time, enabling more efficient optimization.
- Additionally, the findings from the simulation study validate the effectiveness of the FATA-PIDn controller developed for a two-area, three-source LFC system. This conclusion is supported by real-time OPAL-RT-based simulations, confirming the practical applicability of the proposed method in a realistic operational environment.
- Although the present study focused on validating the performance of the FATA-based controllers, future research will include a detailed computational complexity assessment, sensitivity and stability analyses to provide a more comprehensive understanding of system behavior.

## Data Availability

All relevant data are within the manuscript. The collection and analysis method complied with the terms and conditions for the source of the data.

## Abbreviations

2DOF: PDNTwo degrees of freedom proportional derivative with filter
2DOFPID: Two degrees of freedom FPID controller
3DOF-PDN: Three degrees of freedom proportional derivative with filter
ACE: Area control error
ACO: Ant colony optimization
AHB: Artificial humming bird
ALO: Ant-lion optimization algorithm
ANFIS: Artificial neuro fuzzy inference systems
ANN: Artificial neural networks
BB-BC: Big bang-big crunch
BOA: Bear optimization algorithm
CBO: Chaotic butterfly optimization
COA: Cuckoo optimization algorithm
GOA: Grasshopper optimization algorithm
CSOA: Crow search optimization algorithm
DOF-PDN: Degree of freedom proportional derivative with filter
DRLB: Deep reinforcement learning based
DWCA: Discrete water cycle algorithm
ECO: Educational competition optimizer
ESC: Escape optimization algorithm
FATA: Fata morgana algorithm
FHODFC: Fractional high order differential feedback controller
FLPID: Fuzzy logic integrated PID
FLC: Fuzzy logic controller
FOPI: Fractional order proportional-integral
FOPID: Fractional order proportional-integral-derivative
FOPID-PR: Fractional order proportional integral derivative-proportional resonant
FOTID: Fractional order-based tilt-Integral-derivative
FSO: Firebug swarm optimization
GA: Genetic algorithm
GJO: Golden jackal optimization
GWO: Grey wolf optimization
hGWO-PS: Hybrid grey wolf optimization-pattern search
HODFC: High order differential feedback controller
HSCOA: COA into harmony search (hs) algorithm
IAE: Integral of absolute error
ISE: Integral square error
ITAE: Integral of time weighted absolute error
ITSE: Integral of time square error
LMA: Levenberg marquardt algorithm
MAPS: Multi area power system
MFO: Moth flame optimizer
mMSA: Modified moth swarm algorithm
PDN: Proportional-derivative with filter
PI: Proportional-integral
PID: Proportional-integral-derivative
PIDn: Proportional-integral-derivative with filter
PPIDn: Predictive PID with filter
PPIDn: Predictive proportional-integral-derivative with filter
PSO: Particle swarm optimization
PV: Photovoltaic panel
SAR: Search and rescue
SCA: Sine–cosine algorithm
SOA: Skill optimization algorithm
SSA: Salp swarm algorithm
TAPS: Two area power system
TIDDF: Tilt-integral-double derivative filter
TLBO: Teaching learning-based optimization
WCA: Water cycle algorithm
$P\_line12$: Power in the transmission line
V_1_: Voltage of area 1
V_2_: Voltage of area 2
δ_1_: Phase angle of area 1
δ_2_: Phase angle of area 2
X_12_: Impedance of the transmission line
$$f$$: System frequency
ΔP_L_: Instantaneous load changes
Δf_1_: First area frequency change
Δf_2_: Second area frequency change
Δf_tie_: Connection point frequency change
*N*: Series cells per string
λ: Constant coefficient and depends upon the cell material
V_PV_: Cell output voltage
I_SC_: Cell short circuit current
I_PV_: Cell output current
I_PH_: Photocurrent
*M*: Parallel strings
I_0_: Reverse saturation current
R_S_: Series resistance of cell
a: negative value of zero in transfer function
b: Gain of PV system
c: Negative values of poles
d: Negative values of poles
K_*gov*_: Governor gain
K_tur_: Turbine gain
K_reh_: Reheater gain
K_ps_: Power system gain
T_gov_: Governor time constant
T_tur_: Turbine time constant
T_reh_: Reheater time constant
T_ps_: Power system time constant
P_wt_: Wind turbine mechanical output power
ρ: Air density
a_s_: Swept area
V: Wind speed
C_p_: Rotor efficiency
TSR: Tip speed ratio
β: Pitch angle of the blade
ω_r_: Rotor speed
D: Blade rotor diameter
K_p1_: Pitch control gain
K_p2_: Hydraulic pitch actuator gain
K_p3_: Data fit pitch gain
K_fc_: Fluid coupling gain
T_p1_: Pitch control time constant
T_p2_: Hydraulic pitch actuator time constant
T_p3_: Data fit pitch time constant
T_w_: Induction generator time constant
K_P_: Proportional gain
K_D_: Derivative gain
K_I_: Integral gain
K_t_: Tilt gain
K_prd_: Predictive term constant
K_h_: Delay time of the predictive term
n: Derivative action filter constant
λ: Fractional integrator order
t: Time
s: Second
